# RNA Polymerase II at histone genes predicts outcome in human cancer

**DOI:** 10.1126/science.ads2169

**Published:** 2025-02-13

**Authors:** Steven Henikoff, Ye Zheng, Ronald M. Paranal, Yiling Xu, Jacob E. Greene, Jorja G. Henikoff, Zachary R. Russell, Frank Szulzewsky, H. Nayanga Thirimanne, Sita Kugel, Eric C. Holland, Kami Ahmad

**Affiliations:** 1 Basic Science Division, Fred Hutchinson Cancer Center, Seattle, WA, USA; 2 Howard Hughes Medical Institute, Chevy Chase, MD, USA; 3 Present address: Department of Bioinformatics and Computational Biology, University of Texas MD Anderson Cancer Center, Houston, TX, USA; 4 Human Biology Division, Fred Hutchinson Cancer Center, Seattle, WA, USA; 5 Molecular Medicine and Mechanisms of Disease PhD Program, University of Washington, Seattle, WA, USA; 6 Present address: Huntsman Cancer Institute, University of Utah Health Sciences Center, Salt Lake City, Utah, USA

## Abstract

Genome-wide hypertranscription is common in human cancer and predicts poor prognosis. To understand how hypertranscription might drive cancer, we applied our FFPE-CUTAC method for mapping RNA polymerase II (RNAPII) genome-wide in formalin-fixed paraffin-embedded (FFPE) sections. We demonstrate global RNAPII elevations in mouse gliomas and assorted human tumors in small clinical samples and discover regional elevations corresponding to *de novo* HER2 amplifications punctuated by likely selective sweeps. RNAPII occupancy at S-phase-dependent histone genes correlated with WHO grade in meningiomas, accurately predicted rapid recurrence, and corresponded to whole-arm chromosome losses. Elevated RNAPII at histone genes in meningiomas and diverse breast cancers is consistent with histone production being rate-limiting for S-phase progression and histone gene hypertranscription driving overproliferation and aneuploidy in cancer, with general implications for precision oncology.

## One-Sentence Summary

Overexpression of S-phase-dependent histone genes predicts tumor aggressiveness and a general whole-arm chromosome loss bias in human cancers.

Global increase in transcriptional output, or hypertranscription, is associated with poor prognosis in cancer (1). However, it is difficult to reconcile promiscuous incremental upregulation of thousands of genes with the presumed direct action of oncogenic transcription factors in activating expression of target genes to drive tissue-specific malignancies. Alternatively, hypertranscription in cancer may be relevant only to the subset of genes for products that are rate-limiting for cell doubling. Histones are rate-limiting for proliferation (2–4), insofar as all newly synthesized DNA must be packaged into nucleosomes in every cell cycle, and the 64 genes encoding all 5 histone subunits must produce ~5% of a human cell’s total protein during S-phase (5). RNA polymerase II (RNAPII) is so abundant over histone genes during replication that S-phase-specific genome-wide depletion of RNAPII at other genes has been observed in mouse embryonic stem cells (4). S-phase-dependent histone mRNAs are not polyadenylated and so are essentially absent from RNA-seq libraries, and the possibility that they drive over-proliferation has been overlooked.

## RNAPII hypertranscription varies between tumors

**Fig. 1.**
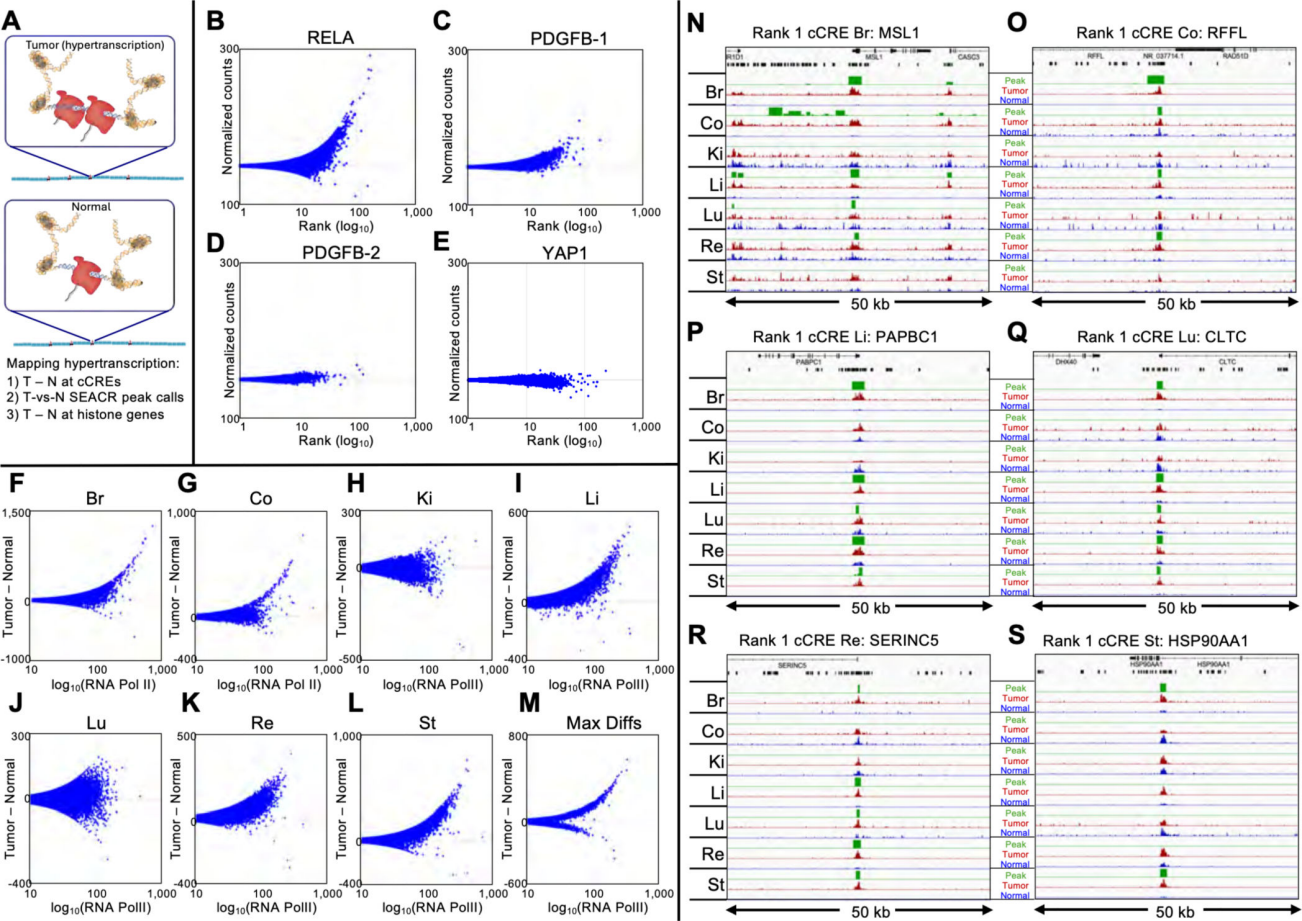
RNAPII-Ser5p FFPE-CUTAC directly maps hypertranscription. (**A**) Model for hypertranscription in cancer: Paused RNA Polymerase II (RNAPII) at active gene regulatory elements such as promoters and enhancers increases on average over the cell cycle, resulting in a net proportional gain in RNAPII occupancy across the genome. Using RNAPII FFPE-CUTAC we can map hypertranscription genome-wide using three complementary approaches: 1) Genome-scaled Tumor (T) minus Normal (N) counts at cCREs, 2) T – N at S-phase-dependent histone genes and 3) SEACR Tumor peak calls using Normal as the background control. (**B-E**) T – N versus log(T + N)/2 plots showing hypertranscription mapped over the 343,731 annotated mouse cCREs for tumor and normal sections dissected post-tagmentation from a 10 micron FFPE slice from each of four different paraffin blocks. Hypertranscription of a cCRE is defined as the excess of RNAPII-Ser5p in the indicated tumor over normal (Tumor minus Normal in normalized count units for Mm10-mapped fragments pooled from the same slide). (**F-M**) All fragments were pooled from four slides from the same paraffin block and the number of fragments equalized between tumor and normal for each of the seven cancers. T – N versus log(T + N)/2 plots showing hypertranscription mapped over the 984,834 annotated mouse cCREs for tumor and matched normal sections from 5 micron FFPE slices. Max Diffs displays the Tumor minus Normal maximum of the seven samples for each cCRE. (**N-O**) For each of the indicated tumors, tracks are shown for 50-kb regions around the #1-ranked cCRE based on Tumor (dark red) and Normal (blue) counts. Raw data tracks were group-autoscaled together for tumor (red) and normal (blue), where SEACR Tumor peak calls (green) use Normal as the negative control. Gene annotations and cCREs (black rectangles) are shown at the top.

We recently adapted Cleavage Under Targeted Accessible Chromatin (CUTAC) for Formalin-Fixed Paraffin-Embedded (FFPE) sections to directly map RNAPII in fixed tissue samples (6). This provides a DNA-based method for measuring transcription, instead of RNA-based methods that are limited by RNA instability and variable transcript half-lives. We assessed RNAPII across 343,731 candidate *cis*-regulatory elements (cCREs) defined by the ENCODE project (7) and compared normal mouse brain tissue to adjacent tumors induced by different transgene drivers (8–10). We observed that significantly upregulated cCREs were more frequent than downregulated cCREs (fig. S1). To sensitively detect RNAPII hypertranscription (Fig. 1A), where the absolute change is important, we first counted the number of mapped fragments spanning each base-pair in a cCRE scaled to the mouse genome coverage and averaged the normalized counts over that cCRE. We then plotted Tumor minus Normal (T-N) counts on the *y*-axis versus the average RNAPII signal (11) on a log_10_ scale on the *x*-axis for clarity. This revealed clear hypertranscription (Tumor >> Normal RNAPII) for the RELA-driven tumor (Fig. 1B). Two PDGFB-driven tumors differed in hypertranscription, high in PDGFB-1 (Fig. 1C) and low in PDGFB-2 (Fig. 1D), whereas the YAP1-driven tumor showed weak hypotranscription (Fig. 1E). To determine whether hypertranscription is specific to any particular class of regulatory element(s), we divided the data into the five ENCODE-annotated cCRE categories and observed that the five RNAPII hypertranscription profiles are highly consistent with one another (fig. S2). This suggests that RNAPII abundance differences between tumors and normal brains affect all regulatory element classes.

We next expanded our findings to a diverse sample of human tumor and matched normal sections from the same patient (fig. S3). We performed FFPE-CUTAC and rank-ordered each pair by Tumor minus Normal (T-N) differences to test for RNAPII hypertranscription based on the 984,834 ENCODE-annotated human cCREs. We observed clear hypertranscription in five of the seven tumors (breast, colon, liver, rectum and stomach) and for the composite of all samples (Fig. 1F–M, fig. S4). In contrast, the kidney and lung tumor samples tested showed essentially no hypertranscription, which implies that hypertranscription is a common, but not a defining feature of cancer (1, 12). We also applied a computational method independent of annotations using the SEACR (Sparse Enrichment Analysis for CUT&RUN) peak caller (13), customized by replacing the background control with the normal sample in each pair. SEACR reported a median of 4,483 peaks that were elevated in tumors, whereas when Tumor and Normal were exchanged only a median of 15 peaks elevated in normal tissue were identified, demonstrating that RNAPII hypertranscription is more common than RNAPII hypotranscription in these cancer samples.

We next asked whether SEACR Tumor-versus-Normal peak calls corresponded to the 100 top cCREs ranked by T-N in the overall list representing all seven tumors. All 100 cCREs at least partially overlapped one or more SEACR Tumor/Normal peak calls, and in addition, the large majority of the 100 top-ranked cCREs intersected with overlapping SEACR peak calls from multiple Tumor/Normal pairs (Table S1). Each of the #1-ranked cCREs in the breast, colon, liver, lung and rectum tumor samples respectively intersected MSL1, RFFL, PABPC1, CLTC and SERINC5 genes and overlapped SEACR peak calls in 4–5 of the 7 tumors (Fig. 1N–S, fig. S5). We conclude that the most strongly RNAPII-hypertranscribed regulatory elements tend to be strongly hypertranscribed in multiple human cancers of different types, including liver cancers from different individuals (fig. S6).

We observed a much lower level of mitochondrial DNA (mtDNA) in most tumor samples than in their matched normal samples for both mouse and human (fig. S7A–B), suggesting that these tumors contain fewer mitochondria. To test this interpretation, we mined publicly available ATAC-seq data from both the TCGA and ENCODE projects and observed similar reductions in cancer samples (fig. S7C–D). Such reductions in mtDNA have been reported based on whole-genome sequencing (14).

## HER2 amplifications with selective sweeps

**Fig. 2.**
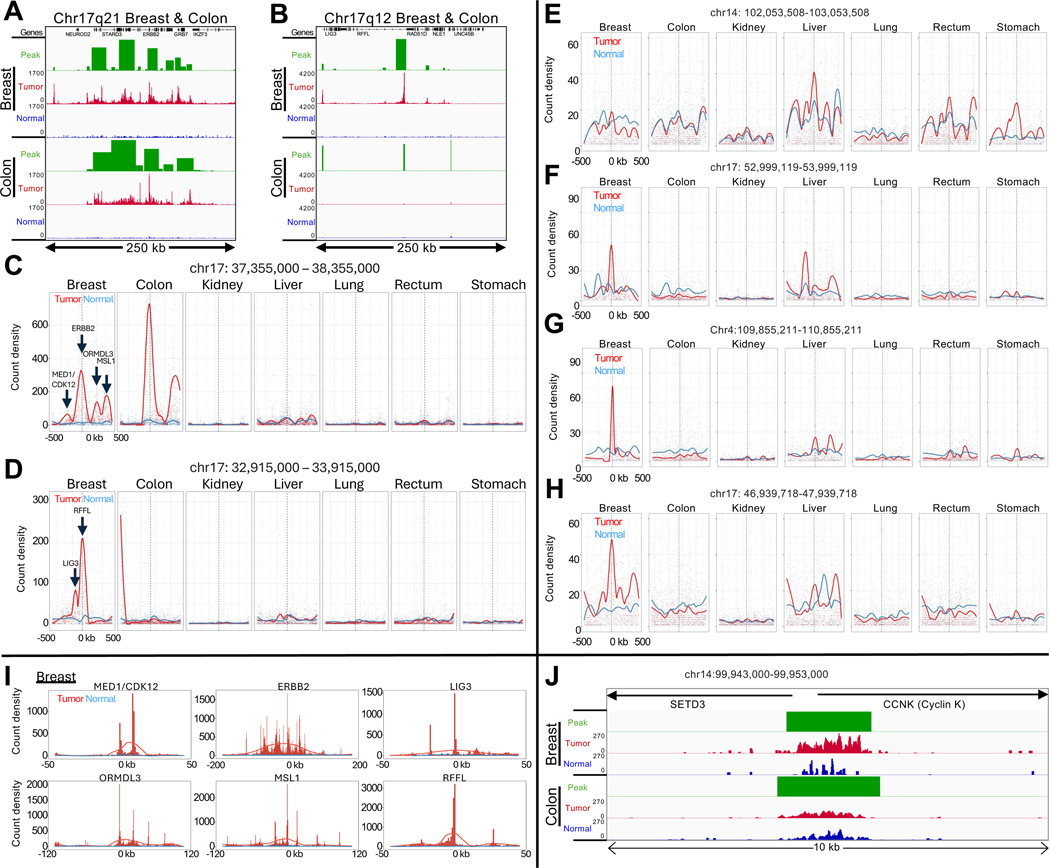
RNAPII levels identify likely HER2 amplifications and regions of linkage disequilibrium. (**A-B**) Normalized count tracks and SEACR peak calls for the 250-kb region on Chromosome 17q21 (A) and for the 250-kb 17q12 region (B) amplified in the breast tumor but evidently not in the colon tumor. Tracks were group-autoscaled together for Tumor (red) and Normal (blue), where SEACR Tumor peak calls (green) use Normal as the negative control. Broad regions of prominent RNAPII hypertranscription indicate likely HER2 amplifications in both tumors. (**C-D**) Bins of 1-kb were tiled over each 1-Mb region centered on the highest peak in Chr17q21 (C) corresponding to the ERBB2 promoter in Chr17q21, and the RFFL promoter in Chr17q12 (D), and count density within each bin is plotted with curve-fitting and smoothing. Each of the six summits in the Breast tumor sample is centered over the promoter peak indicated by an arrow. (**E-H**) Same as (C-D) for top-ranked loci outside of the HER2 amplicon. (**I**) Individual broad summits in (D-E) were zoomed-in and rescaled on the *x*-axis centered over the indicated promoter peak and superimposed over data tracks scaled to the height of the central peak. (**J**) Data tracks for the *CCNK* promoter region, where the normalized count increase in the Breast tumor relative to normal over the 10-kb region shown is 5.4-fold and for Colon is 2.1-fold. The range for the other five tumors is 0.9–2.5 fold.

All but one of the top 25 cCREs are located on Chromosome 17 (Table S1). Eight of these cCREs are within Chr17q12 and 13 are within Chr17q21, each spanning a few hundred kilobases in length in the breast and colon tumors not seen in the normal tissue (Fig. 2A–B, fig. S8). High RNAPII occupancy over the cCREs in Chr17q12–21 can account for most of the RNAPII hypertranscription signal in the breast and colon samples, centered over the ERBB2 gene (fig. S9). ERBB2 encodes human epidermal growth factor receptor 2 (HER2), and is commonly amplified in cancer and is a target of breast cancer therapy (15).

To confirm that the broad regions of RNAPII enrichment around the ERBB2 promoter correspond to HER2 amplifications in the breast and colon patient samples, we applied SEACR, which densely tiled a ~150-kb region centered over the ERBB2 promoter (Fig. 2A–B). To ascertain whether dense tiling using SEACR can detect amplification events, we called SEACR broad peaks on our published K562 RNAPII-Ser5p CUTAC datasets (16, 17). We observed a single region heavily tiled with broad SEACR peaks corresponding to an annotated amplification specific for K562 cells. Zooming in revealed that the end of the densely tiled region corresponded precisely to the t(9;22)(q34;q11) translocation breakpoint of BCR-ABL (fig. S10A–B), which we confirmed by observing a broad SEACR peak on the ABL1 side of the translocation breakpoint (fig. S10C). Thus, our approach using RNAPII-Ser5p CUTAC and SEACR can identify and precisely map regional amplifications such as are found in tumors with BCR-ABL and HER2 amplifications.

To delineate possible RNAPII hypertranscription features within Chr17q12–21, we binned successive 1-kb tiles over each 1-Mb region centered on the highest peak, corresponding to the ERBB2 promoter in Chr17q21 and the RFFL promoter in Chr17q12, and plotted count density within each bin with curve-fitting and smoothing. Multiple broad summits appeared in both breast and colon tumor-versus-normal tracks, and the six summits in the breast tumor sample accounted for the six highest ranked Chr17 promoter peaks (Fig. 2C–D). We similarly plotted count densities of the four highest ranking cCREs outside of Chr17q12–21 (Table S1), but tumor peaks in these regions were at least an order-of-magnitude lower than the ERBB2 peaks in the breast and colon tumor samples (Fig. 2E–H). Of the six summits in the breast tumor sample, ERBB2 and MSL1 also appeared in the colon tumor sample, whereas no other samples showed prominent summits above normal in Chr17q12–21 (Fig. 2C–D). MSL1 encodes a subunit of a histone H4-lysine-16 acetyltransferase complex required for upregulation of the mammalian X chromosome (18).

We next superimposed each of the six summits in the Chr17q12–21 region in the breast tumor sample over the genomic tracks on expanded scales for clarity, centered over the highest promoter peak in the region (Fig. 2I). For ERBB2, the ~100-kb broad summit is almost precisely centered over the ~1-kb wide ERBB2 promoter peak. Although the other summits are less broad, each is similarly centered over a promoter peak. Insofar as there are multiple summits much broader than the promoter peaks that they are centered over, our results are inconsistent with independent upregulation of promoters over the HER2-amplified regions. Rather, it appears that a HER2 amplification event was followed by clonal selection for broad regions around ERBB2 and other loci within each amplicon, consistent with the observation of clonally heterogeneous HER2 amplifications in primary breast tumors by whole-genome sequencing (19). Clonal selection may be driven by selective sweeps (20) following amplification events that generate extrachromosomal DNA in double-minute acentric chromosomes, which partition unequally during each cell division (21–23). Such copy number gains within a tumor can result in intra-tumor heterogeneity (23, 24) and are potential factors for resistance to therapy (25). FFPE-CUTAC thus potentially provides a general diagnostic strategy for detection and analysis of amplifications and clonal selection during cancer progression and therapeutic treatment.

One of the summits in the breast tumor sample absent from the colon tumor sample corresponds to the bidirectional promoters of MED1 and CDK12, both of which have been shown to functionally cooperate with co-amplified ERBB2 in aggressive breast cancer (26, 27). CDK12 is the catalytic subunit of the CDK12/Cyclin K kinase heterodimer complex, which phosphorylates RNAPII for productive transcriptional elongation (28, 29). As Cyclin K is the regulatory subunit of the CDK12 kinase, we would expect that the *CCNK* gene that encodes Cyclin K would be strongly upregulated in the breast tumor but not necessarily in the colon tumor. Indeed, we saw a 5.4-fold increase in RNAPII-S5p over the *CCNK* promoter in the breast tumor relative to adjacent normal tissue, whereas in the colon tumor there is a 2.1-fold increase (Fig. 2J), and a 0.9–2.5-fold increase for the other five tumors, consistent with RNAPII hypertranscription driven in part by CDK12 amplification in this tumor.

## RNAPII over histone genes predicts aggressiveness in meningiomas and breast tumors

**Fig. 3.**
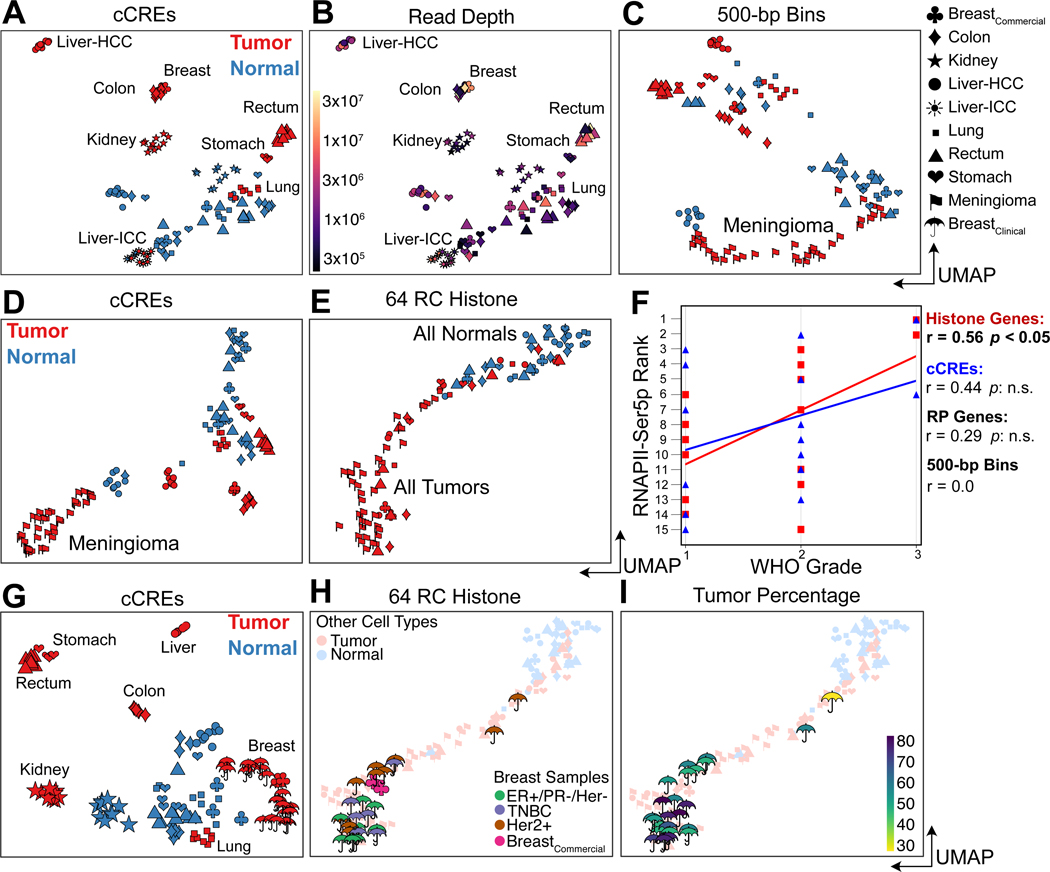
RNAPII over histone genes correlates with aggressiveness in meningiomas (A-F) and breast tumors (G-I). (**A**) UMAP of 114 human tumor samples and replicates including those from 30 meningiomas. HCC: Hepatocellular carcinoma; ICC: Intrahepatic cholangiocarcinoma. (**B**) Same as (A) colored for sequencing depth and indicating homogeneous tumor clusters. (**C**) UMAP of RNAPII FFPE-CUTAC data based on 500-bp bins over the human genome. (**D**) Same as (C) for cCREs. (**E**) Same as (C) for the 64 S-phase-dependent histone genes. (**F**) WHO grade correlates best with RNAPII occupancy over histone genes. (**G**) Same as (A) excluding meningiomas but including samples and replicates from 13 additional breast tumors. (**H**) Same as (E) for breast tumor samples and replicates, colored for tumor type. (**I**) Same as (H) colored for tumor percentage.

To evaluate how effectively FFPE-CUTAC can resolve differences between the seven tumor samples, we constructed a cCRE-based UMAP including 114 individual human datasets. Whereas normal samples produced mixed clusters, tumor samples formed tight homogeneous clusters separated by tissue type (Fig. 3A). This implies that paused RNAPII at regulatory elements is more discriminating between tumors than between the tissues that the tumors emerge from. Relatively few samples and shallow sequencing depths were needed for tight clustering (Fig. 3B).

As the exceptionally S-phase-dependent histone loci are expressed in proportion to the amount of replicated DNA, we wondered whether cancer cell hypertranscription functions to increase engaged RNAPII at these loci to load up on histones at S-phase for more rapid cell proliferation. For both the mouse and human clustered histone genes we observed differences between tumor samples consistent with RNAPII hypertranscription at cCREs differing between samples (fig. S11). If histone production at S-phase is rate-limiting for proliferation, then we would observe a correlation between cancer aggressiveness and RNAPII, specifically over histone genes. As a test of this hypothesis, we applied FFPE-CUTAC to 30 meningioma patient samples and constructed a UMAP that also included the Tumor-Normal pairs described above. Using RNAPII abundance within cCREs, we found that the meningiomas clustered separately from other tumors and from normal (Fig. 3C–D). When we performed the same UMAP construction using only RNAPII abundance over the 64 S-phase-dependent histone genes, we found that all tumors regardless of type clustered together in a cline that overlapped normal at one end (Fig. 3E). This suggested that RNAPII at histone genes can distinguish cancer from normal but is insensitive to cancer type differences. In a double-blind test of whether the cline overlapping normal in multi-dimensional space is an indication of tumor aggressiveness, we rank-ordered the 15 samples with WHO grades based on distance from normal samples for RNAPII occupancy in 500-bp bins, cCREs, histone genes or ribosomal protein genes (Fig. 3F). Best performance was for the histone genes (r=0.57, *p*<0.05), consistent with our hypothesis that high RNAPII levels at histone genes drive proliferation in cancer.

To determine whether S-phase-dependent histone genes can predict cancer aggressiveness in an invasive tumor type, we performed FFPE-CUTAC on a set of 10-μm breast tumor FFPEs from 13 patients representing three major subtypes. When we included these samples with the seven diverse tumor and normal samples (Fig. 1F–L) and used cCREs for UMAP construction, we observed tight clustering according to tumor type (Fig. 3G). However, when we used histone genes for UMAP construction, we observed a single large cluster at one end of a cline that overlapped normal at the other end (Fig. 3H–I). We conclude that histone genes alone can predict cancer aggressiveness in both non-invasive meningiomas and multiple invasive breast cancer subtypes.

## RNAPII over histone genes accurately predicts rapid recurrence in meningiomas

WHO grade is a coarse predictor of recurrence (30), so to predict recurrence for each patient sample, we integrated FFPE-CUTAC data with RNA-seq data using canonical correlation analysis in multi-dimensional space, based on normalized counts over each RefSeq-annotated human gene (fig. S12). We defined a gene as spanning from the 3’-most transcript end through the 5’-most end, stopping when either end of the next gene or LINE element is reached. We observed near-coincidence for 17 of 19 matched RNA-seq and FFPE-CUTAC samples.

**Fig. 4.**
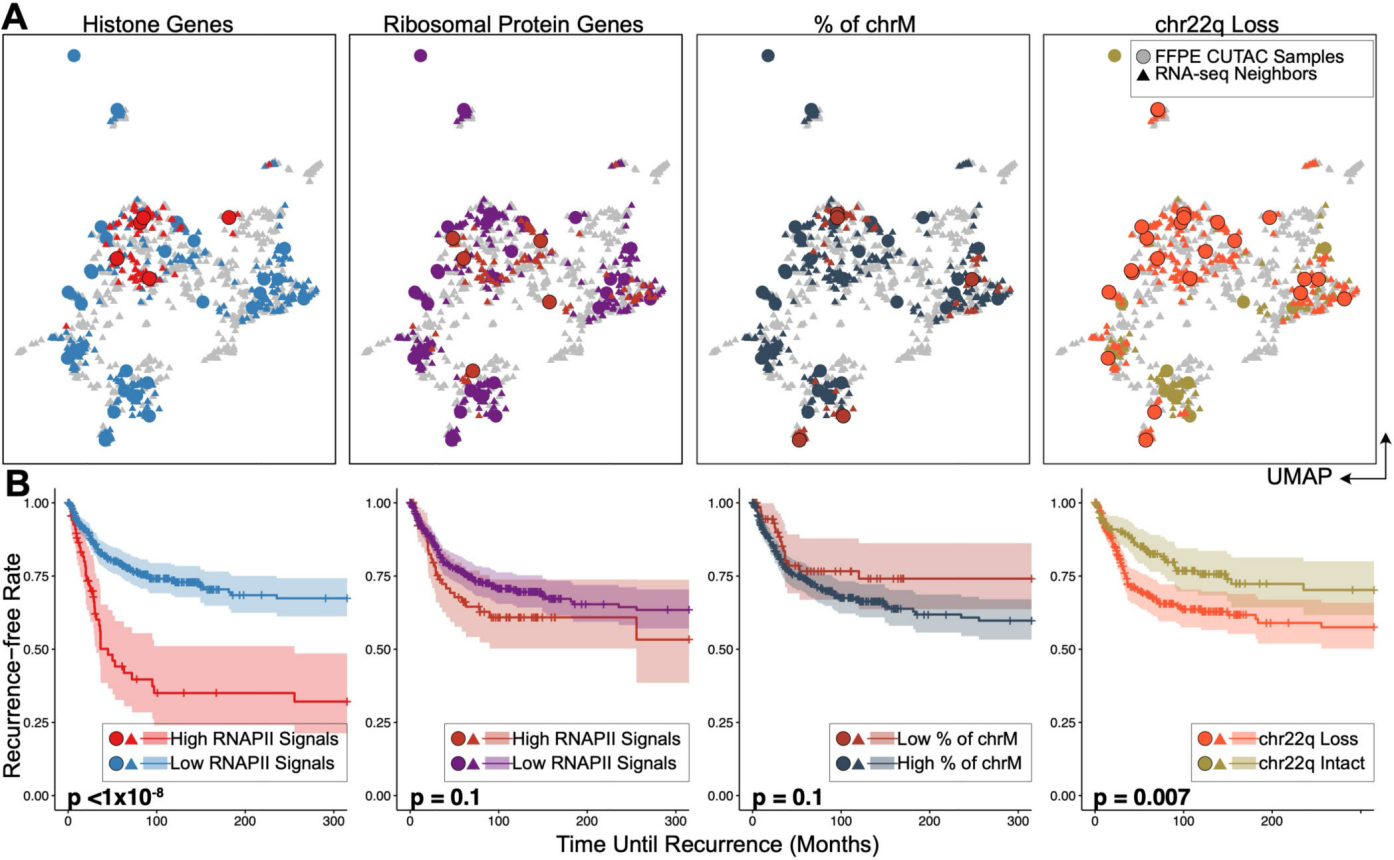
RNAPII over histone genes uniquely predicts recurrence in meningiomas. (**A**) UMAPs of FFPE-CUTAC and frozen RNA-seq meningioma patient samples grouped by different biomarkers. From the left to right: RNAPII over S-phase-dependent histone genes; RNAPII over ribosomal protein genes; Percent of Chromosome M (chrM = Mitochondrial DNA); Chr22q loss. UMAPs are based on integrating FFPE-CUTAC (circles) with frozen-RNA-seq (triangles) samples (fig. S12) using the canonical correlation analysis. For RC histone and ribosomal protein genes, points are colored by high (red) or low (blue for histone genes and purple for ribosomal protein genes) RNAPII enrichment over the corresponding genes for FFPE-CUTAC samples or their shared nearest neighbor RNA-seq samples. For chrM, points are colored by low (red) or high (dark grey) fractions of Chromosome M fragments, with the hypothesis that a low chrM fraction predicts malignant. For chr22q loss, the FFPE-CUTAC samples with chr22q loss and their RNA-seq samples are colored red, while the ones with chr22q intact are colored green. (**B**) Kaplan-Meier (KM) plots of recurrence-free rate as a function of time in months based on each grouping strategy using the biomarkers shown in (C). For fair comparisons between Histone genes, Ribosomal Protein genes and chrM, the same number of samples, i.e., the top five patients, are selected in the malignant group. The fraction of the chrM predictor has settings (6 to 18 samples in the malignant group) with small p values. However, the KM curve order is in the wrong direction for the hypothesis that a low fraction of chrM predicts fast recurrence (fig. S13D).

To predict meningioma patient recurrence, we classified FFPE-CUTAC samples by the histone genes signal and used the top 20 RNA-seq nearest neighbors to compare the patient recurrences (Fig. 4A, fig. S13A). Indeed, we found a highly significant association between high histone signals in FFPE-CUTAC samples and rapid patient recurrence (*p*<10^−8^, Fig. 4B left, fig. S13B). Using only the normalized FFPE-CUTAC counts at the 64 histone genes, we could distinctly separate the five most rapidly recurring from the 25 other tumors which aligns with the generally low recurrence rate of meningiomas, and are predominantly benign (31). In contrast, levels of RNAPII over ribosomal protein genes or mtDNA failed to significantly separate rapidly recurring from benign, regardless of the thresholds applied (Fig. 4B, fig. S13C–D). Although we did observe significant separation of rapidly recurring from benign using Chr22q, the most frequently lost whole-arm (30) (Fig. 4B right), and significant separation for Chr1q gains and Chr6p losses (fig. S13E–F), the levels of significance were much lower than for separation using RNAPII at the histone genes. Our finding that high levels of RNAPII over RC histone genes accurately predicts poor outcomes in meningioma could imply a causal basis.

## RNAPII over histone genes predicts whole-arm chromosome losses

**Fig. 5.**
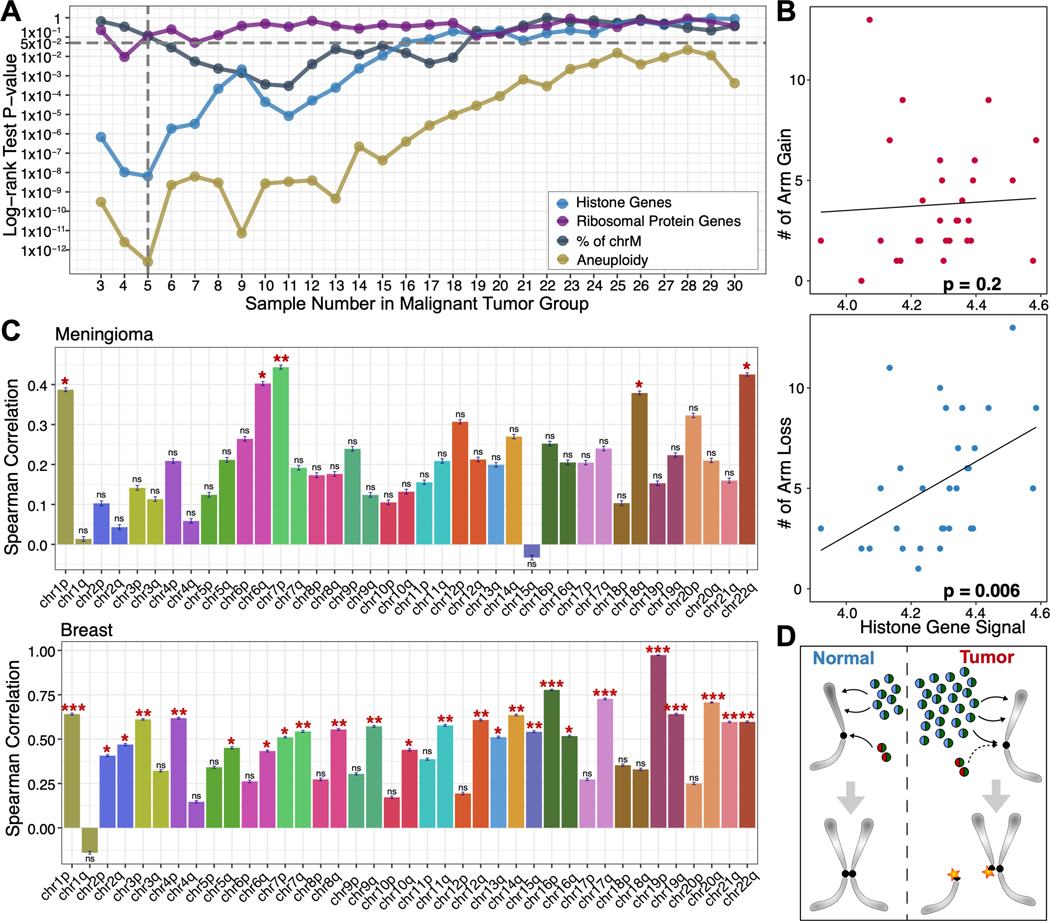
RNAPII over histone genes predicts whole-arm chromosome losses. (**A**) P-values from the log-rank test for the KM separation of malignant from benign for all thresholds of the 30 meningioma patients and the corresponding 36 FFPE-CUTAC samples for Histone genes (blue), Ribosomal protein genes (purple) and % mitochondrial DNA (grey) (Fig. 4), also including the combined aneuploidy data for all 39 chromosome arms (gold). (**B**) Scatterplots and Spearman correlations between RNAPII over histone genes and number of whole-arm gains (upper panel) and losses (lower panel) for each of the 30 meningioma patient samples. (**C**) Spearman correlations between the signal for RNAPII over histone genes and null RNAPII occupancies at cCREs for all 39 autosomal arms for Meningiomas (upper panel) and Breast tumors (lower panel). This summarizes correlation coefficients and significances on the correlation scatterplot in fig. S14. A negative correlation is expected if gains are more frequent than losses (i.e., chromosome arm gain will lead to a lower percentage of cCREs regions with zero RNAPII signals) with increasing RC histone gene signal and a positive correlation if losses are more frequent than gains. The error bar describing the standard deviation of the Spearman correlation coefficient is based on 1000 bootstrap estimation. The significance level is indicated by the stars on the top of each bar with *: p-value < 0.05, **: p-value <0.01, and ***: p-value < 0.001. (**D**) Model for induction of centromere breaks and aneuploidy by histone gene hypertranscription. Histone H3|H4 dimers (blue-green spheres) are deposited along chromosome arms during DNA replication for chromatin duplication, while CENPA|H4 dimers (red-green spheres) are deposited at centromeres and maintain centromere identity. H3|H4 and CENPA|H4 histone dimers are preferentially deposited (black arrows) at the CAF1 and HJURP assembly factors, respectively. However, the excess H3|H4 dimers produced by hypertranscription in cancer cells compete with CENPA|H4 for centromere assembly, reducing centromere function and creating DNA breaks (38, 39). Segregation of broken chromosomes leads to whole-arm aneuploidies.

As a positive control for separation of malignant from benign, we counted total aneuploidies from RNA-seq data and observed best separation for five malignant and 25 benign tumors, closely matching our prediction based on RNAPII at histone genes over the range of 3–7 malignant (Fig. 5A). This led us to ask whether there is a relationship between overproduction of histones and total aneuploidy by plotting RNAPII levels over the histone genes for each patient as a function of the total number of whole-arm chromosome gains or losses. We observed a weak non-significant positive Spearman correlation with gains (p<0.2), but a highly significant correlation with losses (p<0.006) (Fig. 5B). To test whether this excess of losses over gains applied to all chromosome arms, we asked whether for each patient, there was a net increase or decrease in the level of RNAPII over histone genes. Indeed, 38/39 autosomal arms showed a net positive correlation for the meningioma patient population (Fig. 5C, fig. S14A), suggesting that RNAPII at histone genes predicts whole-arm losses in meningioma. We confirmed this excess of whole-arm losses for the breast cancer samples, where we also observed excesses of losses over gains for 38/39 autosomal whole-arm aneuploids (Fig. 5C, fig. S14B), as expected if overexpression of histones drives both over-proliferation and whole-arm chromosome losses in cancer.

## Discussion

We have demonstrated that elevated RNAPII over genes and regulatory elements is a direct measure of hypertranscription in diverse human cancers and identifies and precisely maps amplifications and selective sweeps in small clinical samples. Likewise, we observed a close correspondence between high RNAPII at the 64 S-phase-dependent histone genes consistent with cytological evidence of exceptionally high levels of RNAPII at mouse histone genes (4) and both RNAPII (2, 3) and Myc (32) at Drosophila histone locus bodies exclusively during S-phase. This led us to hypothesize that the single functional role of hypertranscription in cancer is to produce enough histones to keep up with the requirement for packaging new DNA in cancer cells to proliferate faster than normal cells. We confirmed this prediction by performing FFPE-CUTAC on a set of 30 human meningiomas and showing that RNAPII at the 64 S-phase-dependent histone genes, which comprise only 1/100,000^th^ of the human genome, successfully estimated WHO grade and accurately predicted rapid recurrence, which corresponds to elevated expression of proliferation genes (30). Although meningiomas are not invasive, elevated RNAPII at histone genes was also observed in invasive breast tumors. The ability of RNAPII FFPE-CUTAC at only the 64 human genes to predict aggressiveness in a common intracranial tumor and in multiple breast cancer subtypes implies that a rapid PCR assay for histone gene RNAPII or transcription (33) may become an inexpensive general cancer diagnostic tool, revolutionizing precision oncology.

We also found that levels of RNAPII at the S-phase-dependent histone genes correlated with total aneuploidies, which are present in nearly all meningioma patient samples (30), consistent with the occurrence of ~90% whole-arm imbalances in pan-cancer TCGA data (34). Whole-arm losses were observed to be in excess of gains for 38 of the 39 autosomal arms for both meningiomas and breast tumors representing multiple subtypes. To explain how overproduction of histones might account for this striking whole-arm loss bias in patient tumors, we propose that excess H3 histones compete with CENP-A histones at S-phase for nucleosome assembly at centromeres (35, 36) (Fig. 5D). Production of S-phase histones is tightly regulated (37) and overproduction in cancer and displacement of CENP-A nucleosomes are known to result in the generation of DNA–RNA hybrids, likely due to transcription–replication conflicts causing delayed DNA replication, centromere breakage and loss of whole chromosome arms (38, 39). Thus, RNAPII excess over histone genes at S-phase provides a mechanistic basis for understanding not only how cancer cells can proliferate faster than their neighbors but also how this process might generate centromeric breaks and whole-arm imbalances that drive most cancers.

## Supplementary Material

Table S2

Table S1

Supplementary Information

## Data Availability

The sequencing data generated in this study have been deposited in the NCBI GEO database under accession code GSE261351. The raw sequencing data generated from the University of Washington samples are not made available due to data privacy laws. Processed FFPE-CUTAC data for these samples have been deposited on Zenodo and can be accessed with the identifier https://doi.org/10.5281/zenodo.13138686 (40). Custom scripts used in this study are available from https://doi.org/10.5281/zenodo.14649635 (41).
