# Supplementary material for: RNA Polymerase II at histone genes predicts outcome in human cancer
Source: Science. Author manuscript; Available in PMC 2025 Jun 23. (PMC12184985; doi:10.1126/science.ads2169)
Supplement: Supplementary Information [file NIHMS2081179-supplement-Supplementary_Information.pdf]

Science AAAS

Supplementary Materials for

RNA Polymerase II at histone genes predicts outcome in human cancer

Steven Henikoff[1,2,†,*], Ye Zheng[1,3,†], Ronald M. Paranal[4], Yiling Xu[1], Jacob E. Greene[1,5], Jorja G. Henikoff[1], Zachary R. Russell[4], Frank Szulzewsky[4,6], H. Nayanga Thirimanne[4], Sita Kugel[4], Eric C. Holland[4] & Kami Ahmad[1]

Corresponding author: steveh@fredhutch.org

The PDF file includes:

Materials and Methods
Figs. S1 to S14
Tables S1 to S2
References 42-50

Materials and Methods:

Ethical statement

This research was approved by the Fred Hutch Institutional Animal Care and Use Committee (Protocol # 50842) and complies with all required ethical regulations.

Mouse tumor and normal tissues and FFPEs

Ntva;cdkn2a-/- mice were injected intracranially with DF1 cells infected with and producing RCAS vectors encoding either PDGFB (*10*), ZFTA-RELA (*8*), or YAP1-FAM118b (*9*) as has been described (*42*). Upon weaning (~P21), mice were housed with same-sex littermates, with no more than 5 per cage and given access to food/water *ad libitum*. When the mice became lethargic and showed poor grooming, they were euthanized and their brains removed and fixed at least 48 hours in Neutral Buffered Formalin. All animal experiments were approved by and conducted in accordance with the Institutional Animal Care and Use Committee of Fred Hutchinson Cancer Center (Protocol #50842: Tva-derived transgenic mouse model for studying brain tumors). Tumorous and normal brains were sliced into five pieces and processed overnight in a tissue processor, mounted in a paraffin block and 10-micron sections were placed on slides. Mouse tissue (including normal and tumor bearing brains) was removed, fixed in 10% neutral-buffered formalin for a minimum of 24 hours and embedded into paraffin blocks. 10-µm serial sections were cut from formalin-fixed paraffin-embedded specimens and mounted on slides.

Human FFPE slides

The following pairs of human tumor and adjacent normal 5-µm tissue sections on slides from single FFPE blocks were purchased from Biochain, Inc: Breast Normal/Tumor cat. no. T8235086PP/PT; Colon Normal/Tumor cat. no. T8235090PP/PT; Kidney Normal/Tumor cat. no. T8235142PP/PT; Liver Normal/Tumor cat. no. T8235149PP/PT; Lung Normal/Tumor cat. no. T8235152PP/PT; Rectum Normal/Tumor cat. no. T8235206PP/PT; Stomach Normal/Tumor cat. no. T8235248PP/PT. Human primary liver tumor and normal samples were harvested from cases undergoing surgical resection at the University of Washington under the Institutional Review Board approved protocol and then subsequently deidentified.

A set of 31 human meningioma 4-µm tissue sections on slides from the same FFPE blocks used for both frozen-RNA-seq and FFPE-RNA-seq were obtained from the University of Washington as part of a larger study (*30*). An additional set of 15 human breast tumor 10-µm FFPE tissue sections (dates of collection 1999-2012) on slides were obtained from Oregon Health Sciences University Knight BioLibrary. Single slides were subjected to on-slide FFPE-CUTAC and scraped into multiple tubes for PCR to approximately equalize tissue amount between samples. At least one replicate sample from each patient sample provided sufficient data for analysis.

Antibodies

Primary antibodies: RNAPII-Ser5p: Cell Signaling Technologies cat. no. 13523, lot 3; RNAPII-Ser2p: Cell Signaling Technologies cat. no. 13499; H3K27ac: Abcam cat. no. ab4729, lot no. 1033973. Secondary antibody: Guinea pig α-rabbit antibody (Antibodies online cat. no. ABIN101961, lot 46671).

On-slide FFPE-CUTAC

On-slide FFPE-CUTAC was performed as described (*6*) with modifications. The combination of several days in 4% formaldehyde in preparing FFPEs makes the vast majority of the genome impermeable and therefore not recovered. However, the RNAPII-Ser5phosphate epitope, present the 52 tandem copies of the heptapeptide YSPTSPS (364 amino acids without a single lysine) is available, and to a first approximation only over active promoters, enhancers and very highly

expressed genes such as the RC histone genes. Briefly, FFPE slides were placed in 800 mM Tris-HCl pH8.0 in a slide holder and incubated at 85-90°C for 1-14 hours, whereupon the paraffin melted and floated off the slide. Slides were cooled to room temperature and transferred to 20mM HEPES pH 7.5,150mM NaCl. Slides were drained and excess liquid wicked off using a Kimwipe tissue. The sections were immediately covered with 20-60 µL primary antibody in Triton-Wash buffer (20mM HEPES pH 7.5,150mM NaCl, 2mM spermidine and Roche complete EDTA-free protease inhibitor) added dropwise. Plastic film was laid on top to cover and slides were incubated ≥2 hr incubation at room temperature (or overnight at ~8°C) in a moist chamber. The plastic film was peeled back, and the slide was rinsed once or twice by pipetting 1 mL Triton-Wash buffer on the surface, draining at an angle. This incubation/wash cycle was repeated for the guinea pig anti-rabbit secondary antibody (Antibodies Online cat. no. ABIN101961) and for pAG-Tn5 preloaded with mosaic end adapters (Epicypher cat. no. 15-1117 1:20), followed by a Triton-Wash rinse and transfer of the slide to 10 mM TAPS pH 8.5. Tagmentation was performed in 5mM $MgCl_2$, 10mM TAPS pH 8.5, 20% (v/v) N,N-dimethylformamide in a moist chamber and incubated at 55°C for 1 hr. Following tagmentation, slides were dipped in 10 mM TAPS pH 8.5, drained and excess liquid wicked off. Individual sections were covered with 2 µL 10% Thermolabile Proteinase K (TL ProtK) in 1% SDS using a pipette tip to loosen the tissue. Tissue was transferred to a thin-wall PCR tube containing 2 µL TL ProK using a watchmaker's forceps, followed by 1 µL TL ProtK and transfer to the PCR tube. Tubes were incubated at 37°C for 30 min and 58°C for 30 min before PCR as described above.

FFPE-CUTAC for curls

Curls were transferred to a 1.7 mL low-bind tube (Axygen cat. no. MCT-175-C), which tightly fits a blue pestle (Fisher cat. on. 12-141-364). Mineral oil (200 µl) was added and the tube was placed in a 85-90°C water bath for up to 5 min to melt the paraffin. The suspension was then homogenized ~10-20 sec with a pestle attached to a pestle motor (DWK Life Sciences cat no. 749540-0000). Warm cross-link reversal buffer (200 µl 800 mM Tris-HCl pH8.0) was added followed by addition of 6 µl of 1:10 Biomag amine paramagnetic beads (48 mg/ml, Polysciences cat. no. 86001-10). Homogenization was repeated, and 800 µl warm cross-link reversal buffer was added. Tubes were incubated at 85-90°C for 1-14 hours, vortexed, centrifuged briefly and the mineral oil was removed from the top without disturbing the surface. A 500 µl volume of mineral oil was added, mixed by inversion, centrifuged and the mineral oil removed leaving a thin oil layer. A 2.4 µl volume of agarose glutathione paramagnetic beads (Fisher cat. no. 88822) was added below the surface and mixed by inversion on a Rotator. Tubes were centrifuged briefly, placed on a strong magnet (Miltenyi Macsimag separator, cat. no. 130-092-168), and the supernatant removed and discarded, and the bead-bound homogenate was resuspended in up to 1 mL Triton-wash buffer (20 mM HEPES pH 7.5, 150 mM NaCl, 0.5 mM spermidine, 0.2mM EDTA, 0.05% Triton-X100 and Roche EDTA-free protease inhibitor) and divided into PCR tubes for antibody addition. Other steps through to library preparation and purification followed the standard FFPE-CUTAC protocol (*6*). Detailed step-by-step protocols for both slides and curls are available on Protocols.io: https://www.protocols.io/edit/cutac-for-ffpes-c5huy36w.

DNA sequencing and data processing

The size distributions and molar concentration of libraries were determined using an Agilent 4200 TapeStation. Barcoded CUTAC libraries were pooled at equal volumes within groups or at approximately equimolar concentration for sequencing. Paired-end 50x50 bp sequencing on the Illumina NextSeq 2000 platform was performed by the Fred Hutchinson Cancer Center Genomics Shared Resources.

Data analysis

Preparation of the cCREs

We obtained the mm10 and hg38 versions of the Candidate *cis*-Regulatory Elements (cCREs) by ENCODE (https://screen.encodeproject.org/) from UCSC (*43*). For mouse mm10 we used all 343,731 entries. Because our sequencing data was aligned to hg19, we used UCSC's liftOver tool to re-position the hg38 cCREs, resulting in 924,834 entries. We noticed that many human cCREs were in repeated regions of the genome, so we intersected the hg19 cCRE file with UCSC's RepeatMasked regions using bedtools 2.30.0 (*44*) "intersect -v" command to make a file of 464,749 cCREs not in repeated regions.

Preparation of histone regions

For mm10 we used these regions:

chr13	21715711	21837530	H2bc13-H4bc2
chr13	22035122	22043658	H2ac12-H2bc11
chr13	23531044	23622558	H4c8-H1f4
chr13	23683473	23764412	H2ac6-H1f1

For hg19 we used the 64 annotated regions within the two histone clusters (**Table S2**):

chr1	149783434	149859466	Minor
chr6	26017260	26285727	Major

FFPE CUTAC samples alignment of PE50 Illumina sequencing

1. We used cutadapt 2.9 (*45*) with parameters `*-j 8 --nextseq-trim 20 -m 20 -a AGATCGGAAGAGCACACGTCTGAACTCCAGTCA -A AGATCGGAAGAGCGTCGTGTAGGGAAAGAGTGT -Z*` to trim adapters from 50bp paired-end reads FASTQ files.
2. We used Bowtie2 2.4.4 (*46*) with options `*--very-sensitive-local --soft-clipped-unmapped-tlen --dovetail --no-mixed --no-discordant -q --phred33 -I 10 -X 1000*` to map the paired-end 50bp reads to the mm10 Mus musculus or hg19 Homo sapiens reference sequences obtained from UCSC.
3. We used samtools 1.14 (*47*) `*view*` to extract properly paired reads from the mm10 alignments into BED files of mapped fragments.
4. We computed the fraction of fragments mapped to chrM.
5. We used bedtools 2.30.0 `*genomecov*` to make a normalized count track which is the fraction of counts at each base pair scaled by the size of the reference sequence so that if the counts were uniformly distributed across the reference sequence there would be one at each position.
6. We ran Picard 2.18.29 `*MarkDuplicates*` program (http://broadinstitute.github.io/picard/) on the SAM output files from bowtie2.

RNA-seq public samples re-alignment

We re-analyzed 1298 meningioma patient samples, which included raw FASTQ files and clinical outcomes, obtained from Ref. (*31*). For alignment, we used the reference genome hg19 from GENCODE (v19). The RNA-seq data was aligned using STAR version 2.7.11 with the following parameters for per-sample 2-pass mapping and we utilized the unstranded RNA-seq counts as the raw count per gene per sample:

--outSAMtype BAM Unsorted
--outSAMattributes NH HI NM MD AS nM
--twopassMode Basic
--twopass1readsN -1

--quantMode TranscriptomeSAM GeneCounts
--readFilesCommand zcat

Preparation of aligned samples

1. For mouse on-slide samples, we merged tumor replicates within the experiment. The raw count was normalized with respect to the sequencing depth and genome coverage prior to generating the genome browser track visualization and conducting the hypertranscription analysis.
2. For BioChain human on-slide tumor and normal samples, we merged mapped fragments from different experiments for each tumor or normal. We then equalized the numbers of fragments for tumor and normal pairs by downsampling the larger set of the two using the UNIX `*shuf*` command. This ensured that each matching tumor-normal sample comparison was based on the equal total number of reads.
3. Meningioma FFPE-CUTAC samples, with RNAPII enrichment counts taken at 500bp genomic binning, cCRE regions, and genes respectively, were normalized by the term frequency-inverse document frequency (TF-IDF) normalization method (*48*) to remove the sequencing depth biases.

Peak calling

We ran SEACR 1.3 (*13*) with parameters `*norm relaxed*` on tumor samples with the normal sample from each tumor and normal pair as the control. For comparison, we also called peaks after reversing the roles of tumor and normal.

Preparation of and Tumor-Normal file per cCRE region

We used the bedtools `*intersect*` and `*groupby*` commands to sum normalized counts within the cCRE region boundaries. The resulting files have one row per cCRE region and one column per sample and are suitable for submission to the Degust server (https://degust.erc.monash.edu/) using the `*Voom/Limma*` option (-$\log_{10}$FDR versus $\log_2$FoldChange). We computed Tumor-Normal pairs from the cCRE region files and sorted them by largest differences in absolute value (**Table S1**).

Curve-fitting

We partitioned the genome into 1 kb tiles and merged replicates, then downsampled to equalize library sizes between tumor and normal samples from each patient and added up normalized counts within each tile. For each tumor and normal patient sample, we fit the normalized counts across tiles using a Local Polynomial Regression (LOESS) model as implemented in the `*stats*` package of the R programming language, setting the degree of smoothing to 0.2 specified by the `*span*` parameter of `*loess*` function.

UMAPs

To ensure the quality of samples for downstream analysis, we excluded BioChain tumor or normal samples with fewer than 100,000 read counts or less than 10,000,000 bp of total fragment length. We utilized 500bp genomic binning, cCRE regions, or gene regions as the genomic features, respectively. Specifically, each gene feature was defined as the region spanning from the 3'-most transcript end through the 5'-most end, stopping when either end of the next gene or

LINE element s reached. For histone gene features related analysis, we took the 64 corresponding histone gene regions from the all-gene regions defined as above. We calculated the raw sequencing read count overlapping each type of feature regions using the `*getCounts*` function from the chromVAR R package. Processing the feature regions by samples count matrix, we initially applied the TF-IDF normalization method (*48*). This method first normalizes read counts across samples to correct for differences in total read depth and then adjusts across cCRE regions, assigning higher values to rarer regions. TF-IDF normalization was implemented using the `*RunTFIDF*` function from the Signac R package. This step was followed by the selection of top features using `*FindTopFeatures*` from the Signac package and data scaling performed by `*ScaleData*` from the Seurat package. Subsequently, we conducted principal component analysis (PCA) on the scaled data and then used the top 50 principal components to generate a UMAP representation, providing a refined visualization of the relationship across samples.

Meningioma WHO grade prediction

All 64 gene features are used to generate the UMAP to demonstrate the separation power of histone gene between tumor and normal samples. The average distance to all the normal samples are calculated on 50 dimensional latent embeddings using Euclidean distance for each tumor sample. The FFPE-CUTAC meningioma samples were then ranked based on the distance to normal samples, with longer distance ranked higher and shorter distance ranked lower. This ranking was subsequently correlated with the corresponding WHO grade of meningioma patients.

FFPE-CUTAC and RNA-seq meningioma samples integration

We leveraged the canonical correlation analysis strategy (*48*) to integrate the FFPE-CUTAC samples with the public RNA-seq meningioma data. First, we quantified the FFPE-CUTAC RNAPII enrichment signals across genes to obtain a gene-by-sample count matrix in the same format as the RNA-seq processed data. Both FFPE-CUTAC and RNA-seq data were normalized by log-transformation of counts per million (CPM), center-scaled, and subjected to principal component analysis (PCA) on the top variable features. For the integration, we retained almost all the genes using the top 5,7000 features and the top 250 PCs for dimension reduction. The `FindIntegrationAnchors` and `IntegrateData` functions from the Seurat v5.1 R package were used to find the anchor samples between FFPE-CUTAC and RNA-seq, and to integrate two modalities. We set `k.anchor = 5` to find the top 5 matching samples in the other modality. The integrated UMAPs were generated using 250 PCs and 15 nearest neighbors, with the remaining parameters set to default values.

Meningioma recurrence prediction

We defined the overall RNAPII signals across histone genes by taking the median of the TF-IDF normalized counts of 64 histone genes. The density distribution across 30 meningioma patients provided the general pattern of histone signals (**fig. S11A**). Considering that meningioma is mostly benign, we set the threshold at 4.4 to call a high histone signal at the right tail of the highest density peak. Five patients are hence deemed to have high RNAPII enrichment at histone genes for the prediction of faster recurrence. For a fair comparison, the top five patients were also used for the ribosomal protein genes and the fraction of Chromosome M (chrM) prediction (**Fig. 4B-D**).

We next investigated whether the high histone signal could predict recurrence outcomes. Due to the lack of clinical records for the 30 meningioma patients profiled by FFPE-CUTAC, we leveraged public frozen RNA-seq data of meningioma, which includes recurrence time and status

information. These RNA-seq data have been successfully integrated and constructed into a shared nearest neighbor graph with our FFPE-CUTAC samples. The top 20 nearest neighbors were collected for each of the FFPE-CUTAC patient samples, and the Kaplan-Meier curves were generated for high and low histone signal groups, respectively. Log-rank test was used to compare the survival curves and determine if the recurrence rate in the high-histone-signal population was significantly faster than the low-histone-signal group. We implemented the same analysis on ribosomal protein genes and the fraction of chrM as negative controls and also explored the prediction performance using a series of thresholds to define the malignancy group.

Aneuploidy prediction of meningioma recurrence

CaSpER was used to detect chromosome arm gain or loss for meningioma patients through the matching RNA-seq data. We followed the CaSpER tutorial (https://github.com/akdess/CaSpER?tab=readme-ov-file) (*49*) demonstrated using the meningioma data from the Yale study (*50*). We used the same control samples, i.e., SRR3996005, SRR3996002, SRR3995998, SRR3996001, SRR3995989, SRR3995987, SRR3996004 and SRR3995995, from GEO: GSE85133. The same default parameter value, 0.75, was used when calling chromosome arm gain or loss using the function ` extractLargeScaleEvents` from the CaSpER R package.

Fig. S1.

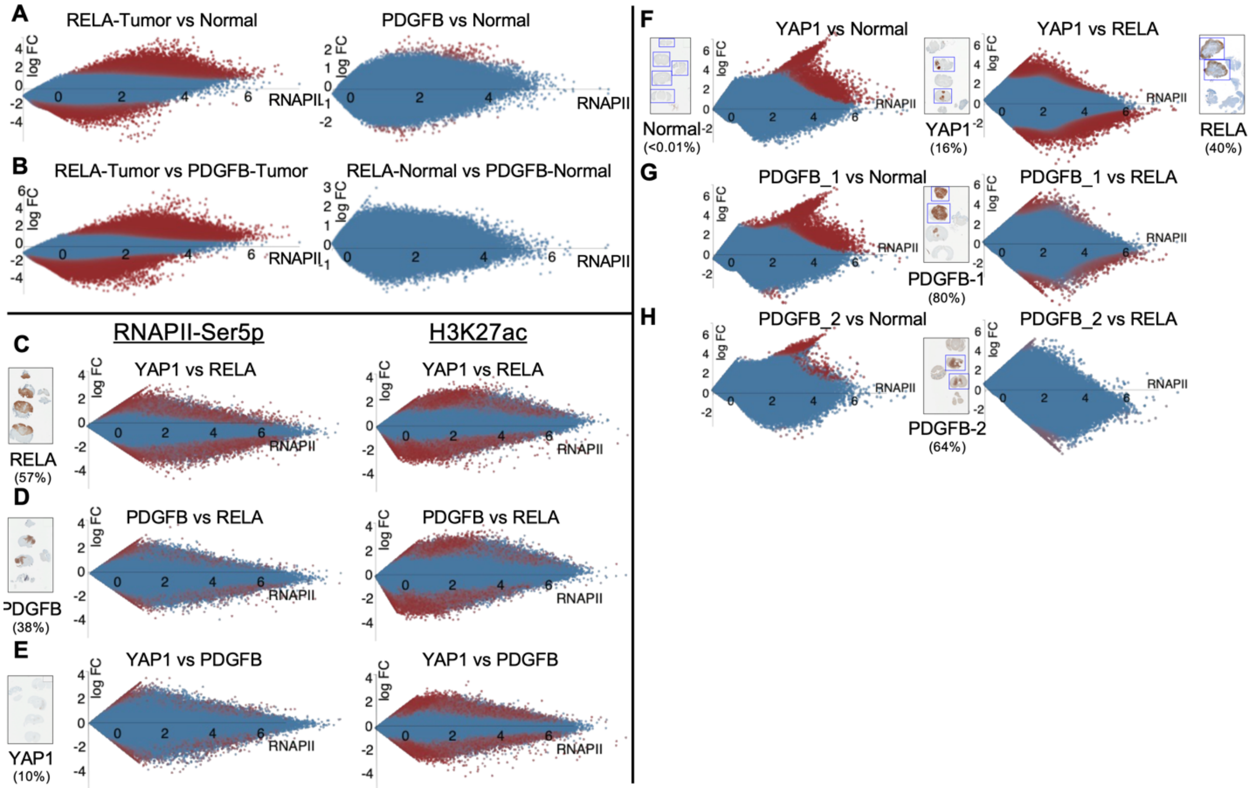

RNAPII-Ser5p FFPE-CUTAC shows stronger and more frequent changes in upregulation than downregulation of cCREs. The Voom/Limma option of the Degust server (https://degust.erc.monash.edu/) was applied to mouse cCRE RNAPII-Ser5p FFPE-CUTAC data from pooled replicates from 5 RELA and 4 PDGFB experiments. MA plots display $x = \log_{10}(\text{Tumor*Normal})/2$ versus $y = \log_2(\text{Tumor/Normal})$ for normalized counts from the tumor and normal samples being compared, and red color indicates FDR < 0.05. Normalized counts are the fraction of counts at each base pair scaled by the size of the Mm10 reference sequence (2,818,974,548), so that if the counts are uniformly distributed across the reference sequence, there would be one at each position. (**A-B**) Both RELA and PDGFB tumor sections show higher counts than normal sections but significant RELA changes both up and down are far stronger than PDGFB changes, confirmed in a head-to-head comparison between tumors and normal sections. (**C-E**) Same as (A-B) except using either RNAPII-Ser5p or histone H3K27ac antibodies for FFPE-CUTAC and using entire 10-µm curls divided into 4-8 samples per curl for PCR and sequencing. For MA plots, data were merged from multiple experiments and equalized by downsampling to 10 million fragments, with 4 merged replicates per sample. DAP-stained slides for each paraffin block used, with the total fraction of tumor indicated in parentheses. (**F-H**) Voom/Limma was used to construct MA plots based on individual 10-µm sections from single slides corresponding to the boxed sections on slides DAP-stained for tumor-driver transgene expression. Numbers in parentheses are percentages of tumor cells based on the numbers of stained and unstained cells within the boxed sections. Relative to normal, more cCREs increase in RNAPII than decrease.

Fig. S2.

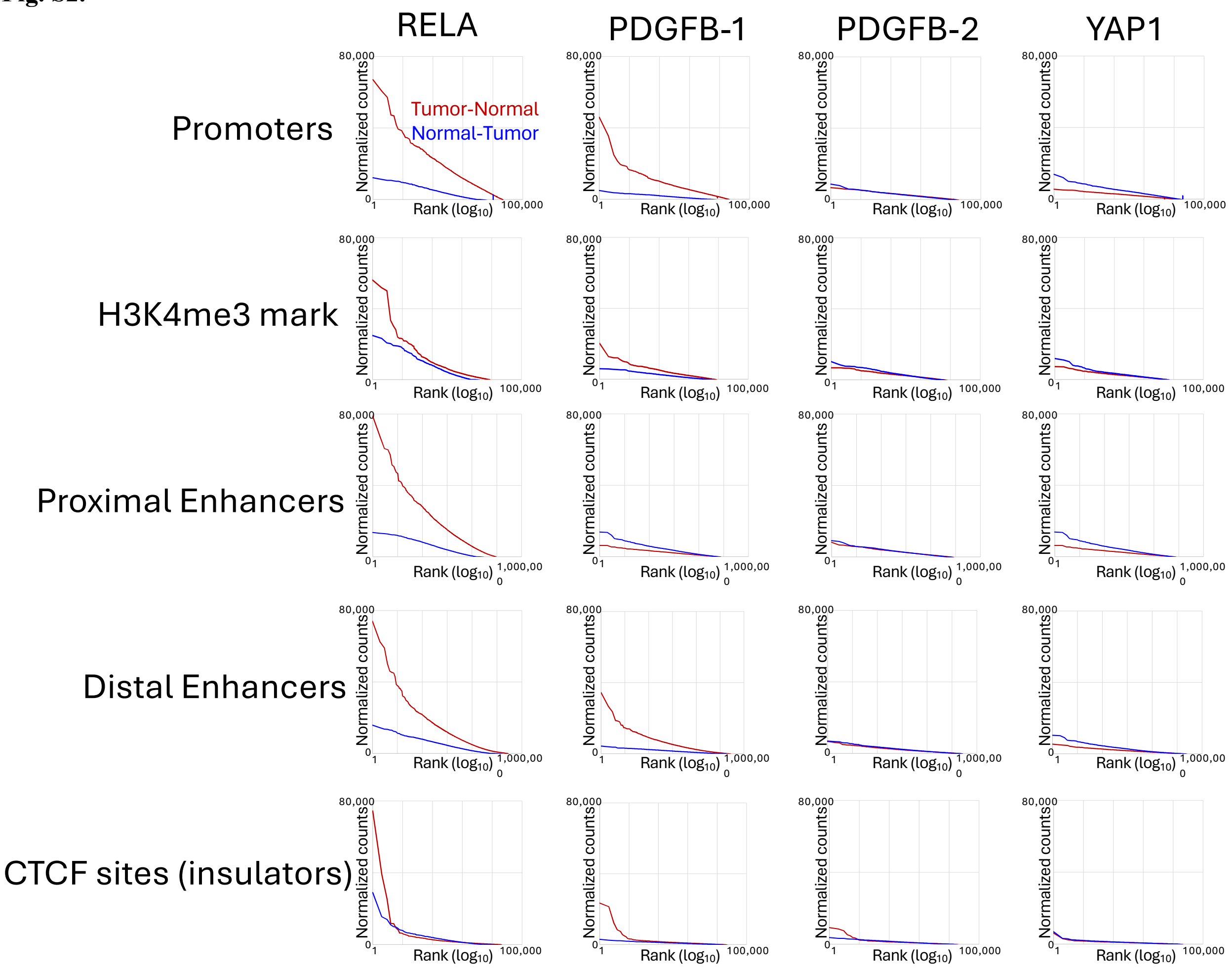

Hypertranscription mapped over the 343,731 ENCODE-annotated mouse cCREs categorized by regulatory element type. For each tumor and normal sample, we counted the number of mapped fragments spanning each base-pair in a cCRE scaled to the mouse genome and averaged the number of counts over that cCRE across tumor or normal samples. We then divided up the 343,731 cCREs into the five ENCODE-annotated categories: Promoters (24,114), H3K4me3-marked cCREs (10,538), Proximal Enhancers (108,474), Distal Enhancers (211,185) and CTCF cCREs (24,072) and rank-ordered based on tumor minus normal representing global upregulation (red). Conversely, we rank-ordered cCREs based on normal minus tumor representing global downregulation (blue). With such a large collection of loci, our a priori expectation is that the rank-ordered distribution of differences between tumor and normal will be approximately the same regardless of whether the differences are based on tumor minus normal or normal minus tumor. For clarity, we plotted rank-ordered differences on a $\log_{10}$ scale. Strong hypertranscription for RELA and PDGFB-1, weak hypertranscription for PDGFB-2, and little or no hypotranscription for YAP1, are seen for all classes, consistent with the T – N versus $\log_{10}$(T + N)/2 plots shown in Figure 1B-E.

Fig. S3.

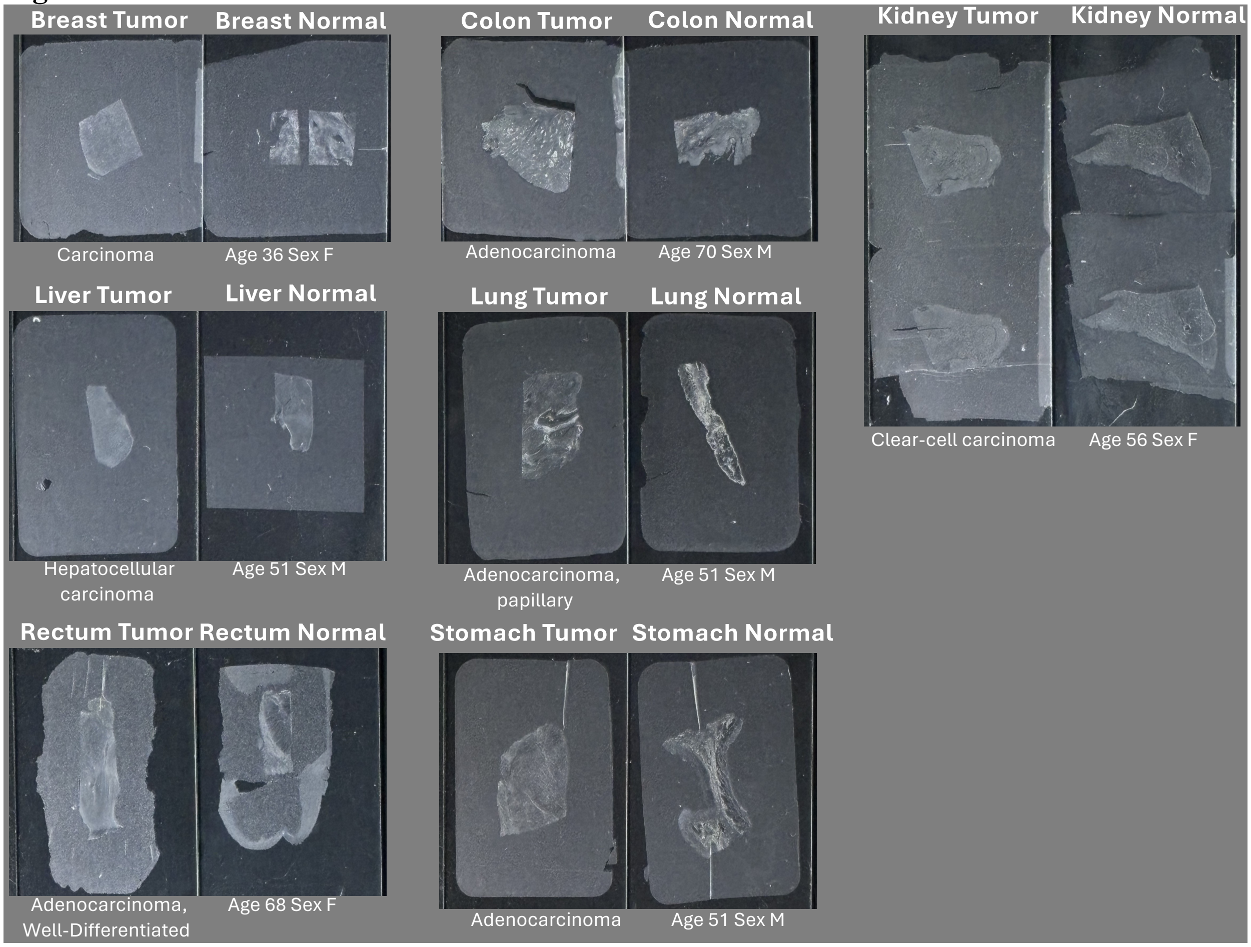

Photographs of 5-µm FFPE sections from human tumor and adjacent normal tissues. Pathology classification, age and sex were provided by the vendor (BioChain). Each image spans the width of a standard charged microscope slide, where the tissue is visible under the paraffin skin. On-slide RNAPII-Ser5p FFPE-CUTAC was applied to slides in parallel, using a total of four slides each for 100 separate samples in all to produce the data analyzed in this study.

Fig. S4.

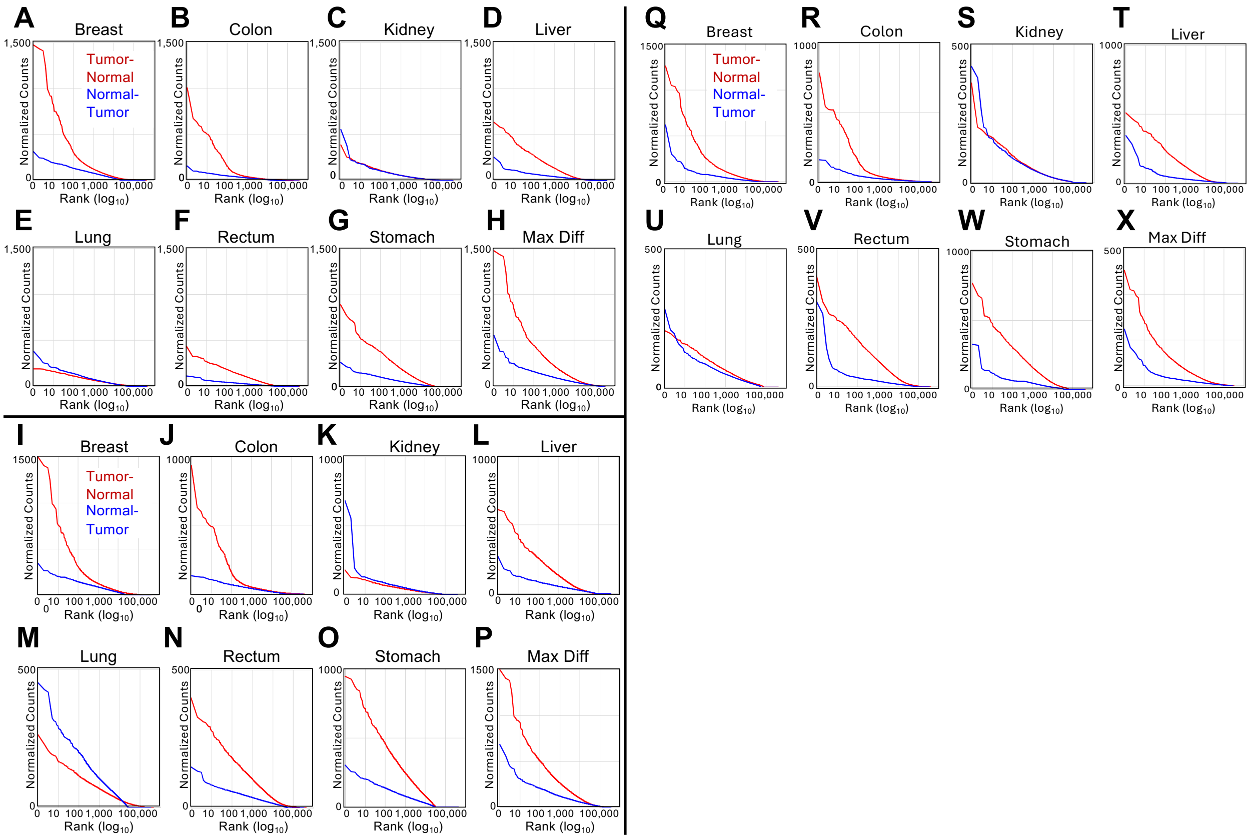

Hypertranscription in human Tumor-vs-Normal tissues. Related to Figure 2. (**A-H**) Same data as in Figure 2A-H, except plotted as in Figure S2 to facilitate comparisons. (**I-P**) Combined data from a single slide with duplicate removal. (**Q-X**) Combined data from four slides after removing duplicates and equalizing the number of fragments between tumor and normal sections. The number of unique fragments per sample in each Tumor/Normal pair is that Breast: 1,125,608; Colon: 3,712,097; Kidney: 2,031,893; Liver: 2,983,411; Lung: 1,123,638; Rectum: 3,284,736; Stomach: 719,598.

Fig. S5.

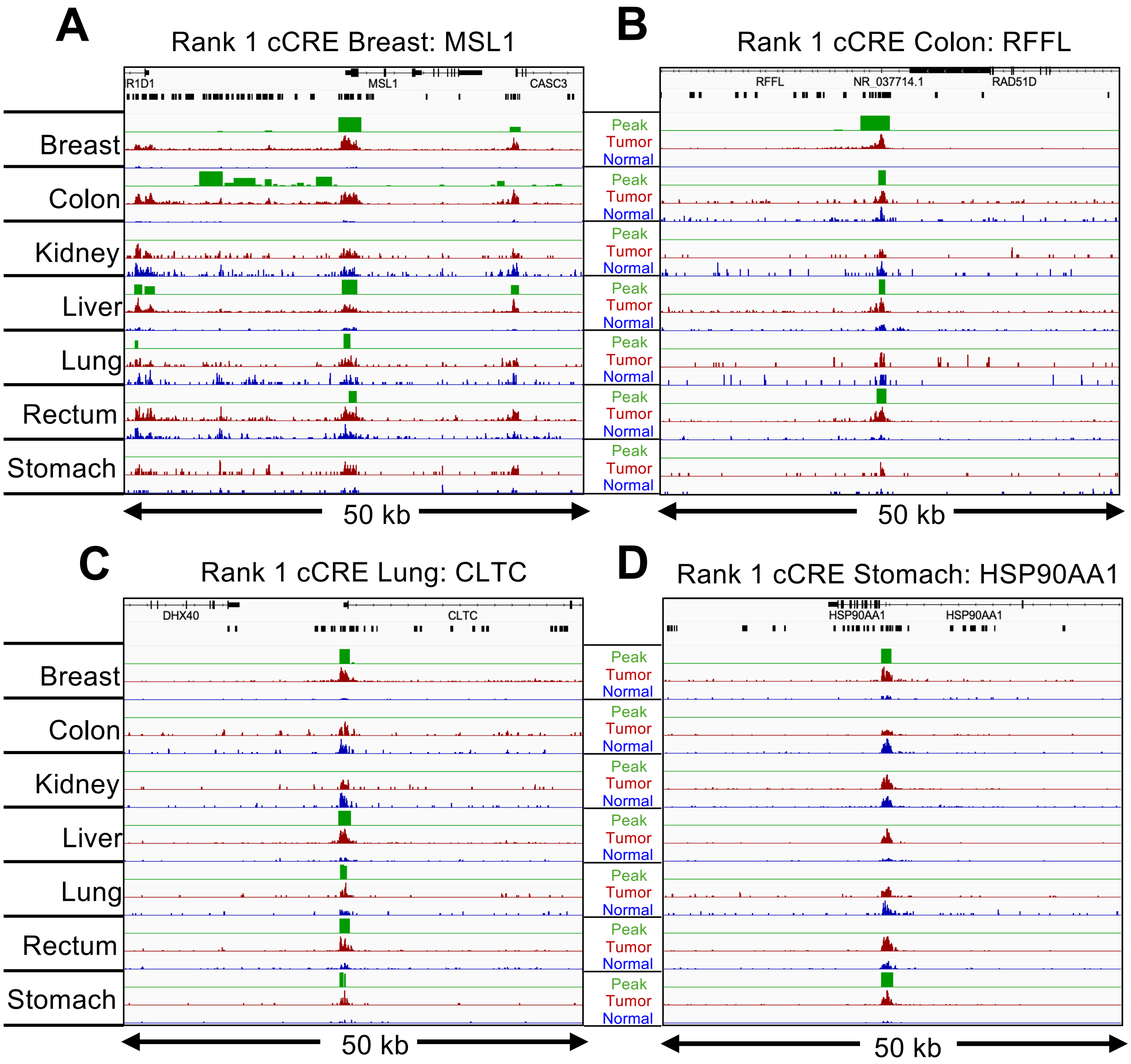

Genome browser tracks for the top-ranked CRE regions for Breast, Colon, Lung and Stomach. For each of the indicated tumors, tracks are shown for 50-kb regions around the #1-ranked cCRE based on Tumor (dark red) and Normal (blue) counts. Data tracks were group-autoscaled together for Tumor (red) and Normal (blue), where SEACR Tumor peak calls (green) use Normal as the negative control. Gene annotations and cCREs (black rectangles) are shown at the top. The #1-ranked cCREs intersected promoters in the Breast, Colon and Lung samples and intersected an intergenic enhancer in the HSP90AA1 gene in the Stomach sample.

Fig. S6.

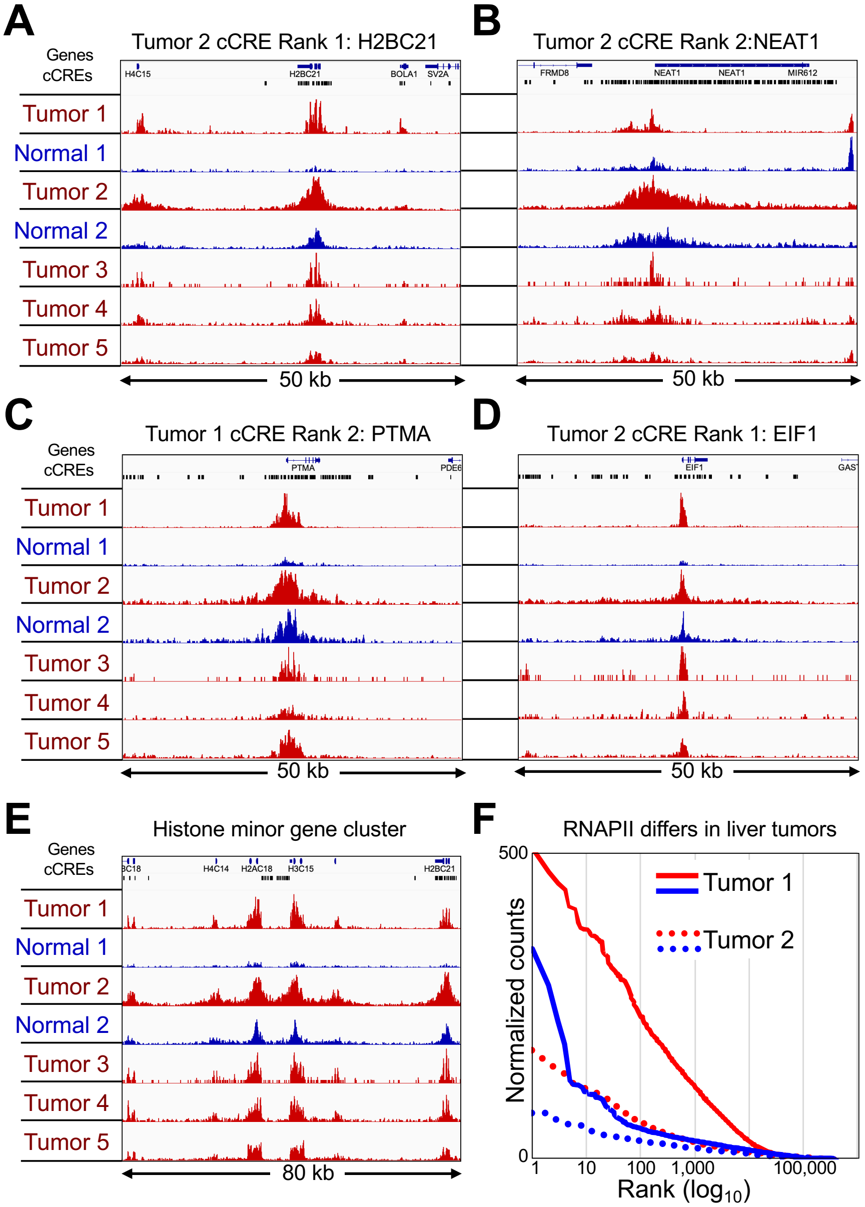

Hypertranscription differs between human liver tumors. (**A-D**) Top-ranked cCREs based on liver tumors 1 and 2 (red) and matched normal (blue) counts. Tumor/Normal tracks and Tumors 3-5 are group-autoscaled. (**E**) Same as (A), except for the minor histone gene cluster on Chromosome 1. (**F**) Levels of hypertranscription differ between different hepatocarcinomas (Tumor 1: solid lines, Tumor 2 dotted lines, where tumor is red and normal is blue). For each tumor and normal sample, we counted the number of mapped fragments spanning each base-pair in a cCRE scaled to the human genome and averaged the number of counts over that cCRE. We rank-ordered based on tumor minus normal representing global upregulation, and conversely rank-ordered cCREs based on normal minus tumor representing global downregulation. With such a large collection of loci, our a priori expectation is that the rank-ordered distribution of differences between tumor and normal will be approximately the same regardless of whether the differences are based on tumor minus normal or normal minus tumor. For clarity, we plotted rank-ordered differences on a $\log_{10}$ scale.

Fig. S7.

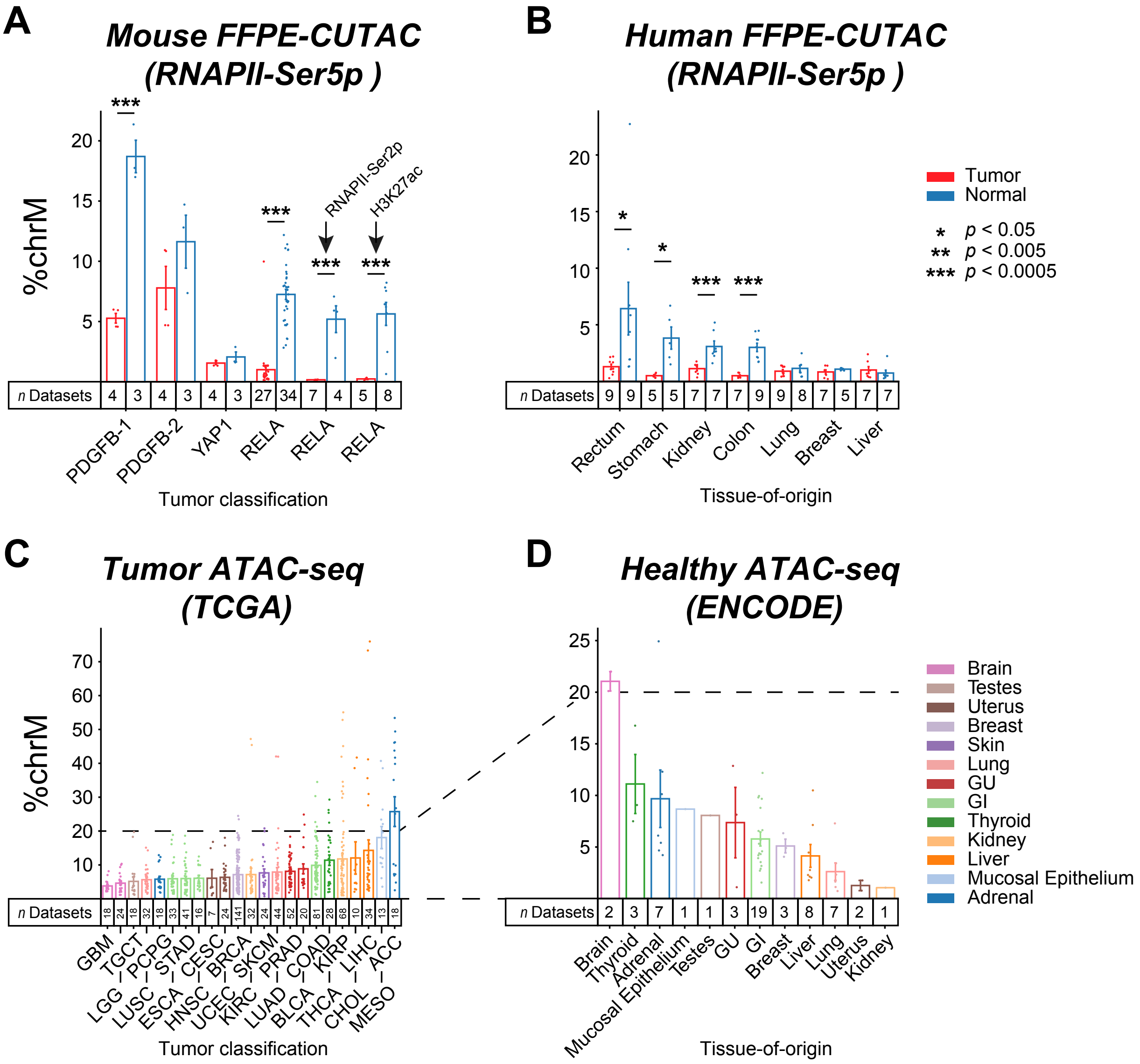

FFPE-CUTAC mitochondrial DNA signal is reduced in tumors. A) The percentage of normalized counts mapping to Chromosome M (chrM = mitochondrial DNA) was calculated for FFPE-CUTAC data from four mouse brain tumor paraffin blocks driven by PDGFB, YAP1 and RELA transgenes. An RNAPII-Ser5p antibody was used for the first four comparisons, and an RNAPII-Ser2p and histone H3K27ac antibodies were used respectively for the fifth and sixth comparisons. (**D**) Same as (C) for RNAPII-Ser5p FFPE-CUTAC data for the seven human Tumor/Normal pairs used in this study. (**E-F**) ATAC-seq count data from TCGA (tumor) and ENCODE (normal) shows variability in chrM percentages between tumors, consistent with our finding based on FFPE-CUTAC.

Fig. S8.

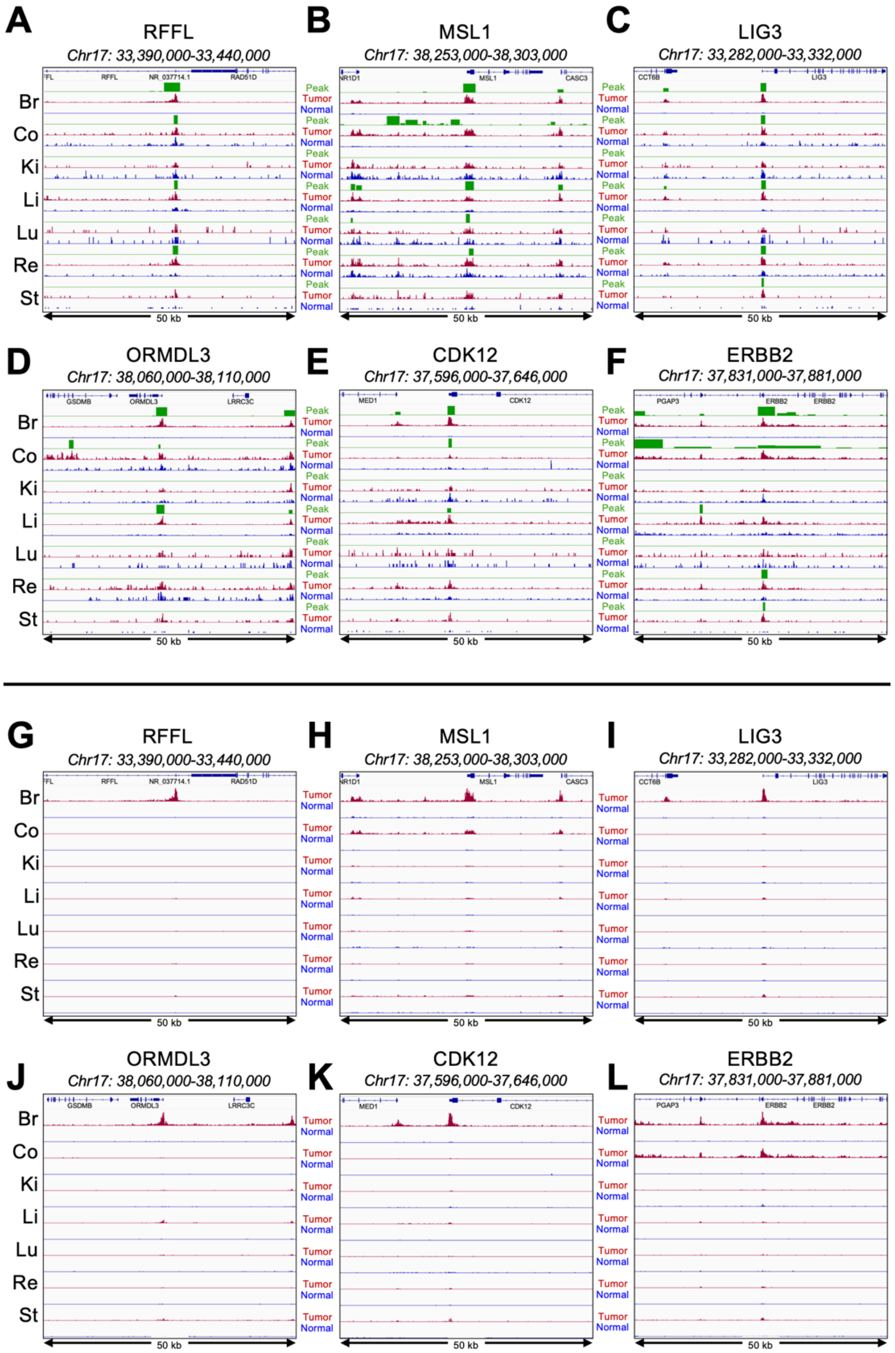

Focal hypertranscribed regulatory elements embedded in broad regions of hypertranscription on Chromosome 17q12-22. (**A-F**) The six most highly transcribed cCREs within the ~5 Mb region of Chromosome 17q1.2-2.2 are displayed with each tumor (dark red) and normal (blue) pair scaled to one another so that peaks can be observed in all samples. SEACR peaks (green) are group-autoscaled in all panels. (**G-L**) Same as (A-F) except that all tumor-normal samples are group-autoscaled to the height of the tallest peak, where the disappearance of all the peaks except for those in Breast and for MSL1 and ERBB2 in Colon is evidence that peaks in these regions are strongly hypertranscribed in Breast and partially in Colon but not in any of the other tumors.

Fig. S9.

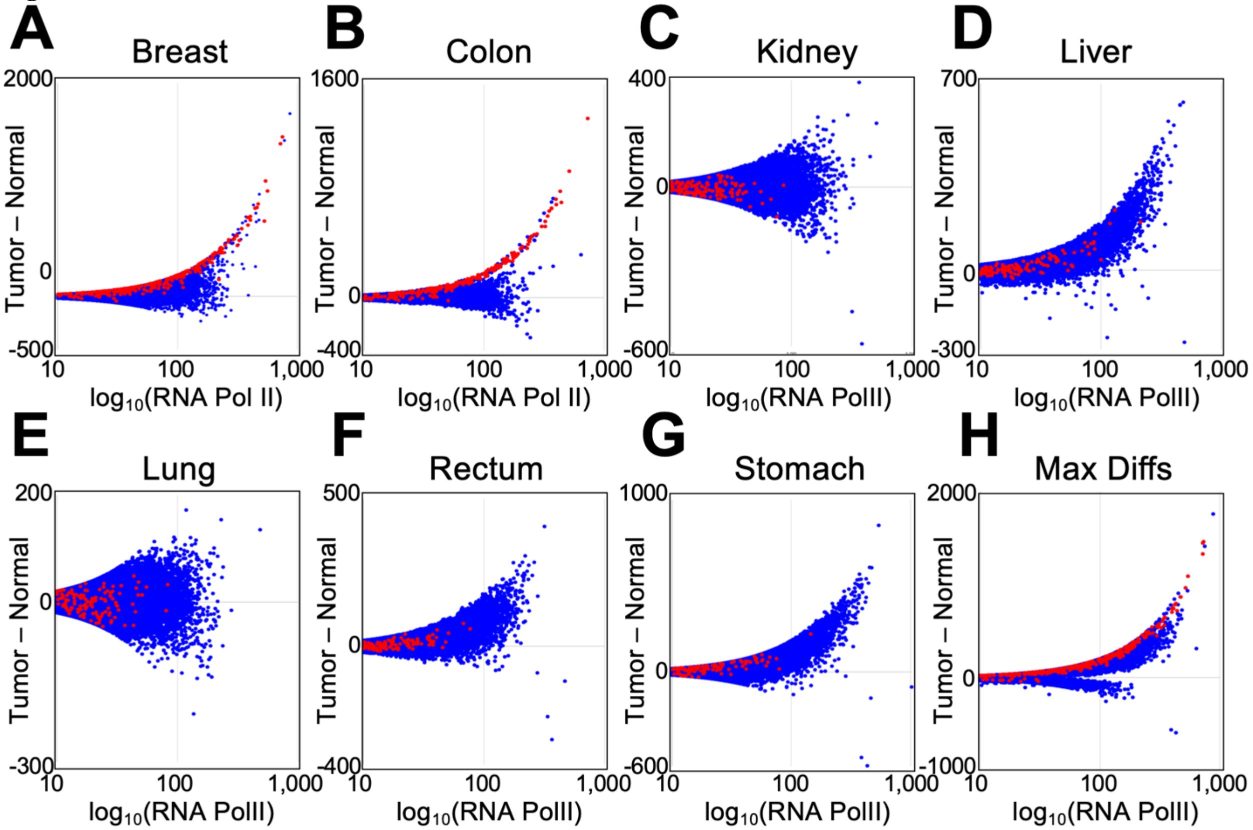

HER2 amplifications account for most of the hypertranscription signal in the Breast and Colon cancer samples. Panels A-H correspond to Figure. 1F-M, where the superimposed red dots are cCREs within Chr17q12-21.

Fig. S10.

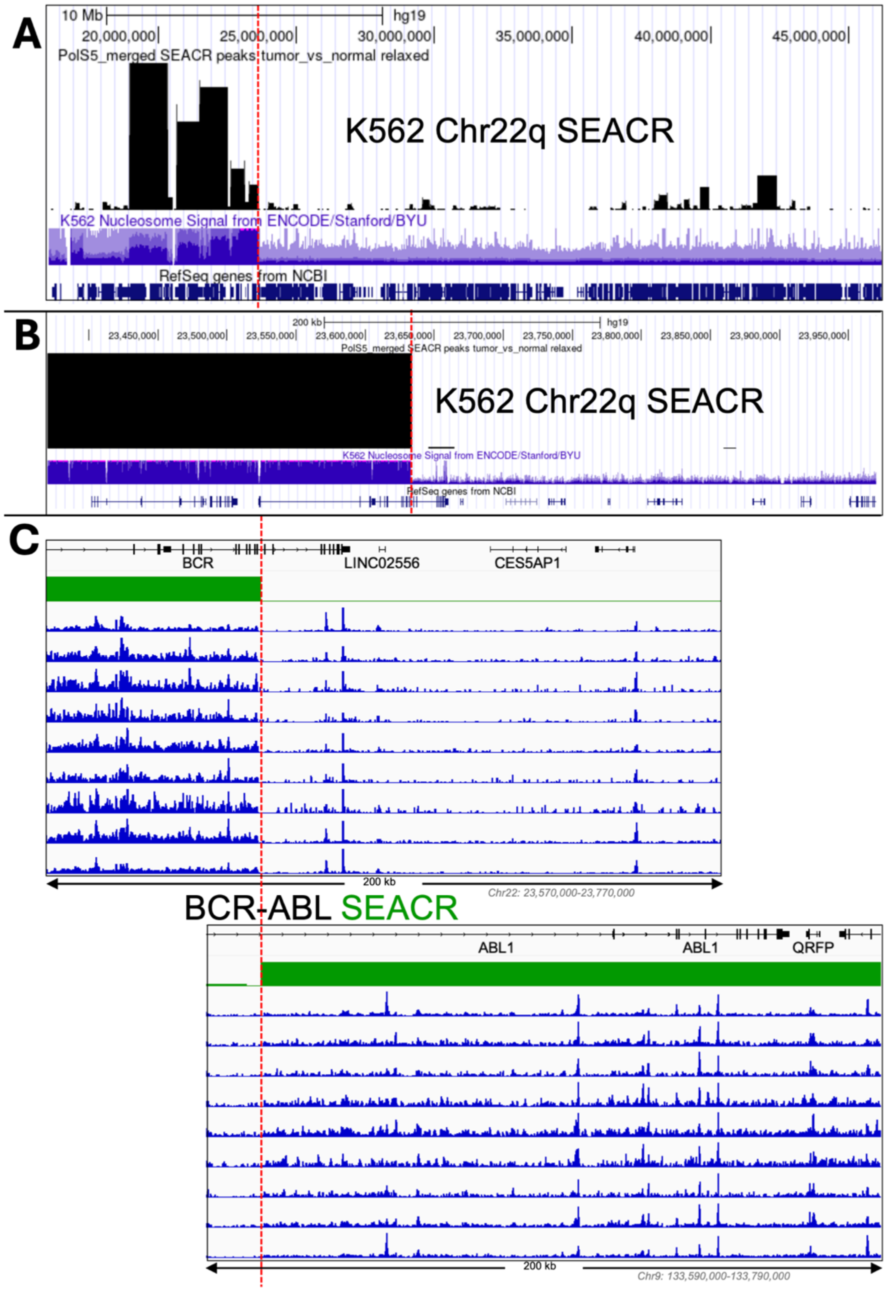

SEACR identifies and precisely maps amplifications in cancer. We called SEACR peaks on a merged set of RNAPII-Ser5p CUTAC datasets from K562 cells, which are annotated for amplifications in human genome build hg19. An amplified region (purple tracks) in K562 cells on Chr22q is shown as a UCSC browser track at (**A**) 25 Mb and (**B**) at 600 kb scales, together with SEACR broad peaks called on published K562 RNAPII-Ser5p CUTAC datasets (*19, 20*). Dotted red line indicates the location of the BCR-ABL t(9;22)(q34;q11) translocation breakpoint on Chr22q. (**C**) The precise correspondence to the annotated amplified regions is evident in the SEACR tracks and in 9 autoscaled tracks from 3 separate experiments, where the breakpoint in each gene (marked by the dotted red line) precisely corresponds to the change in signal amplitude, with abrupt increases within the part of each gene that is amplified, precisely mapped by SEACR.

Fig. S11.

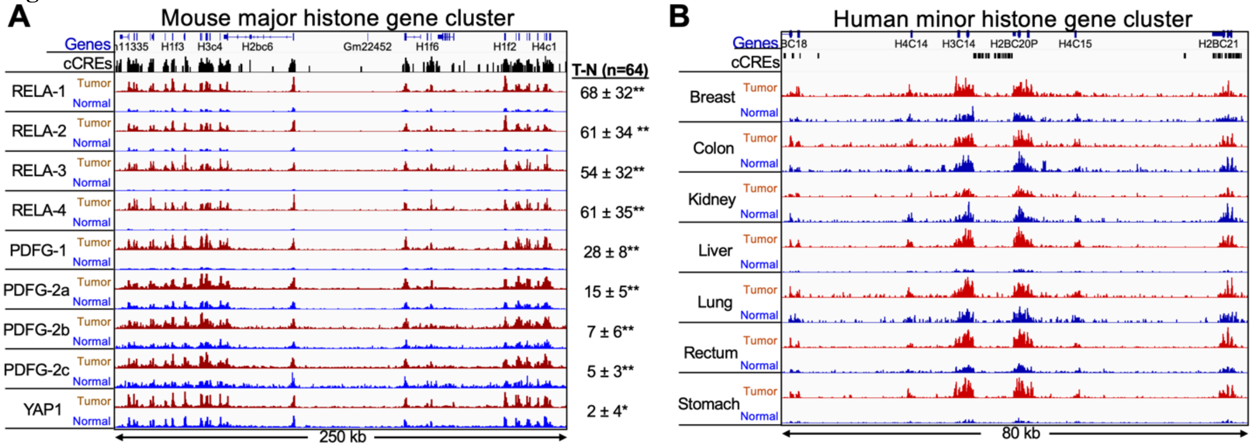

Hypertranscription at S-phase-dependent histone genes. (**A**) IGV tracks of the major histone gene cluster on Chromosome 13 from fusion-transgene-driven mouse brain tumors. Tumor and Normal 10-µm sections were from the same slide. Slides used for PDGFB-2a-c were from the same paraffin block but used in different experiments, and all others were from different paraffin blocks. Numbers at right were obtained by subtracting the sum of normalized counts in the normal sections from that in the tumor sections over all 64 annotated single-exon S-phase-dependent histone genes, where the Standard Deviation is shown. Paired *t*-test: * $p < 0.001$; ** $p < 0.00001$. (**B**) IGV tracks of the human minor histone gene cluster on Chromosome 1, where tracks are autoscaled for each Tumor (red) and Normal (blue). Tumor and Normal 5-µm sections were from a matched pair of slides taken from the same patient.

Fig. S12.

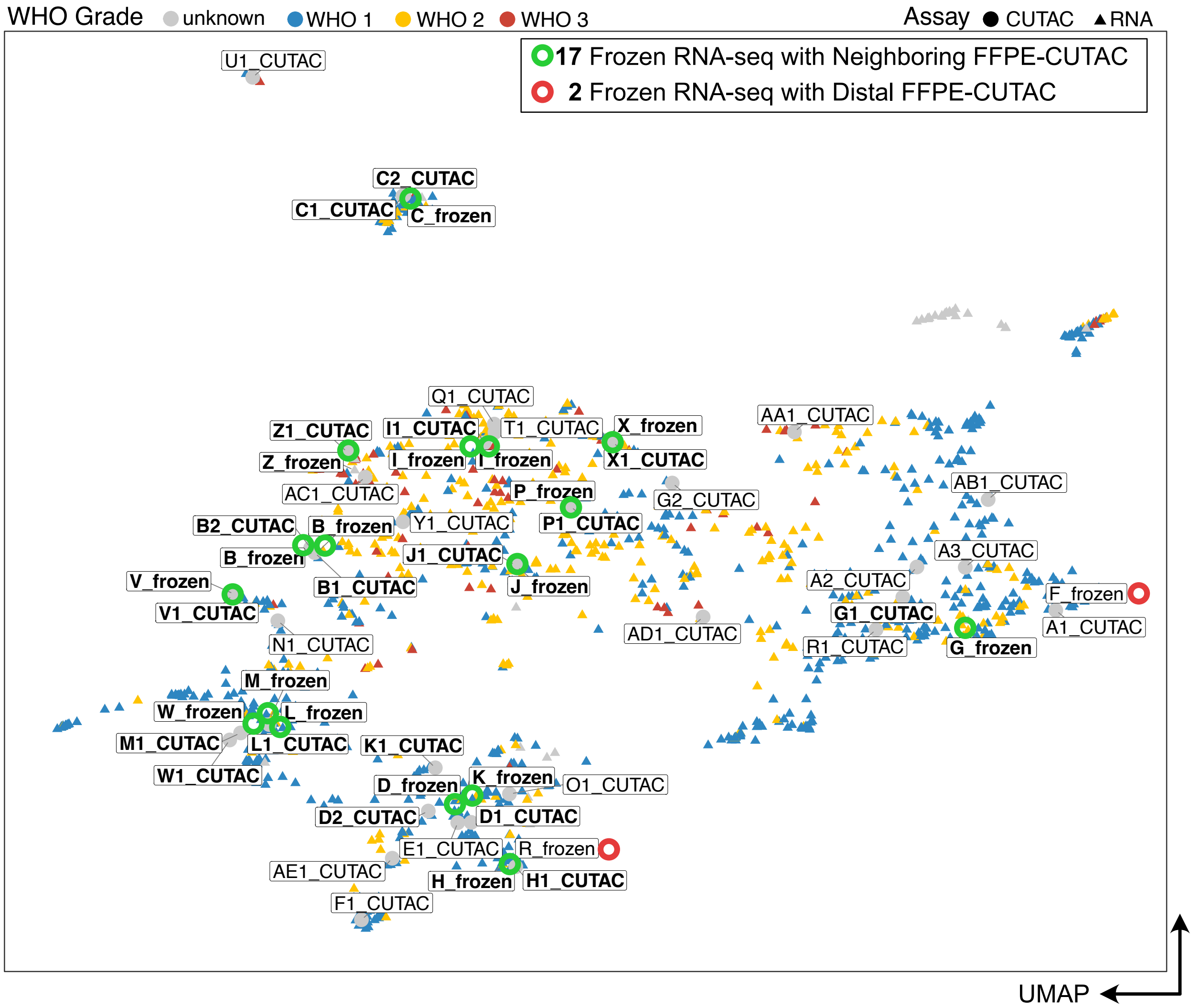

5 **Integration of FFPE-CUTAC samples with frozen RNA-seq meningioma.** Green circles mark frozen RNA-seq samples (triangles) with a close and matched FFPE-CUTAC neighbor (dots), and red circles mark RNA-seq samples without a close matching FFPE-CUTAC neighbor. Points are colored by the WHO grade.

Fig. S13.

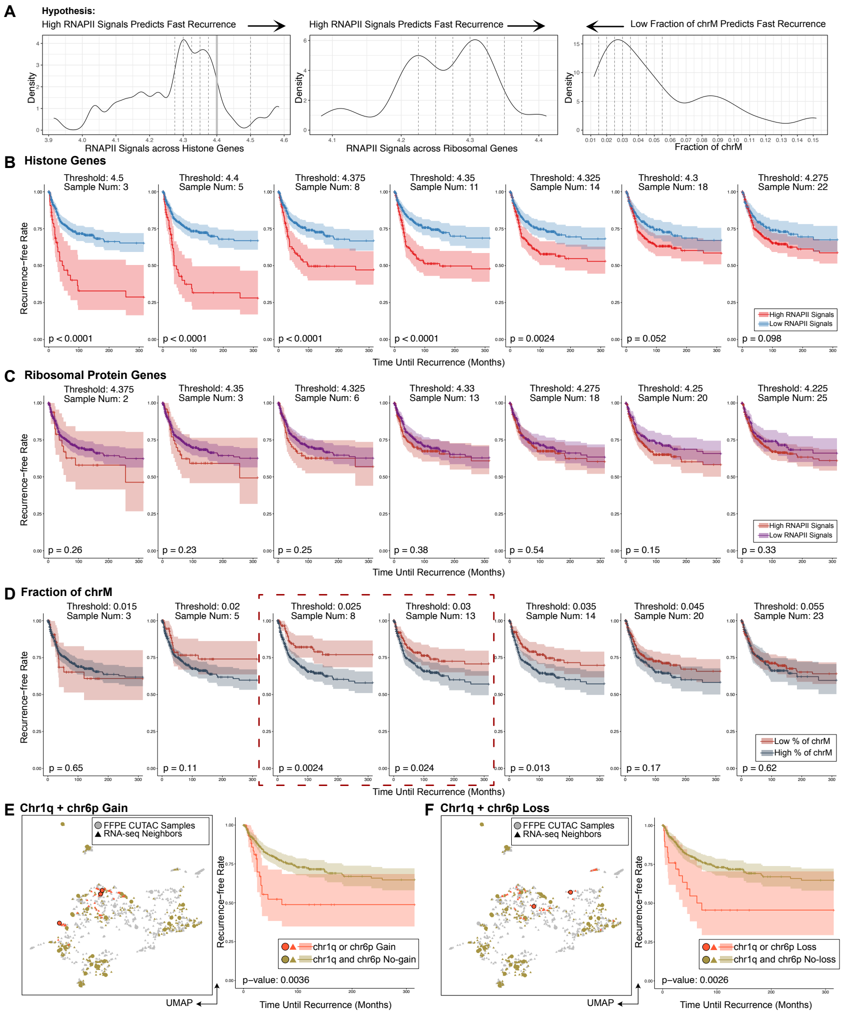

RNAPII at 64 histone genes predicts recurrence time of meningiomas but RNAPII at Ribosomal Protein genes or fraction of mitochondrial DNA do not. (**A**) Density distribution of the RNAPII at Histone genes (left) and Ribosomal Protein genes (middle), and the fraction of mitochondrial DNA (chrM, left) across 30 meningioma patients and 36 FFPE-CUTAC samples. Our hypothesis is that high RNAPII signals or a low fraction of chrM predict faster recurrence. Threshold 4.4 (grey line) in the Histone gene panel is set to separate the malignant from the benign group used in Figure 4, considering most meningioma patients are benign. Dashed lines correspond to a series of separation thresholds to illustrate the survival changing pattern in (B-D). (**B-D**) Kaplan-Meier (KM) curves compare the recurrence between the malignant and benign group predicted by RNAPII signals at 64 S-phase-dependent histone genes (B) and ribosomal protein genes (C) and the fraction of chrM (D). The sample number in each panel title indicates the sample number in the predicted malignant group. The p-values at the left-bottom corner of each panel are the log-rank test evaluating the separation of two survival curves. 95% confidence intervals are shown as ribbons around the curves. The dashed square highlights the significant thresholding settings for chrM, but the KM curve order is against the hypothesis that low chrM predicts malignancy. (**E-F**) Evaluation of the recurrence difference between patients with any chromosome 1q (chr1q) and chromosome 6p (chr6p) gain (E) or any loss (F), where histone genes major cluster is located on chr1q and the minor cluster is located on chr6p.

Fig. S14.

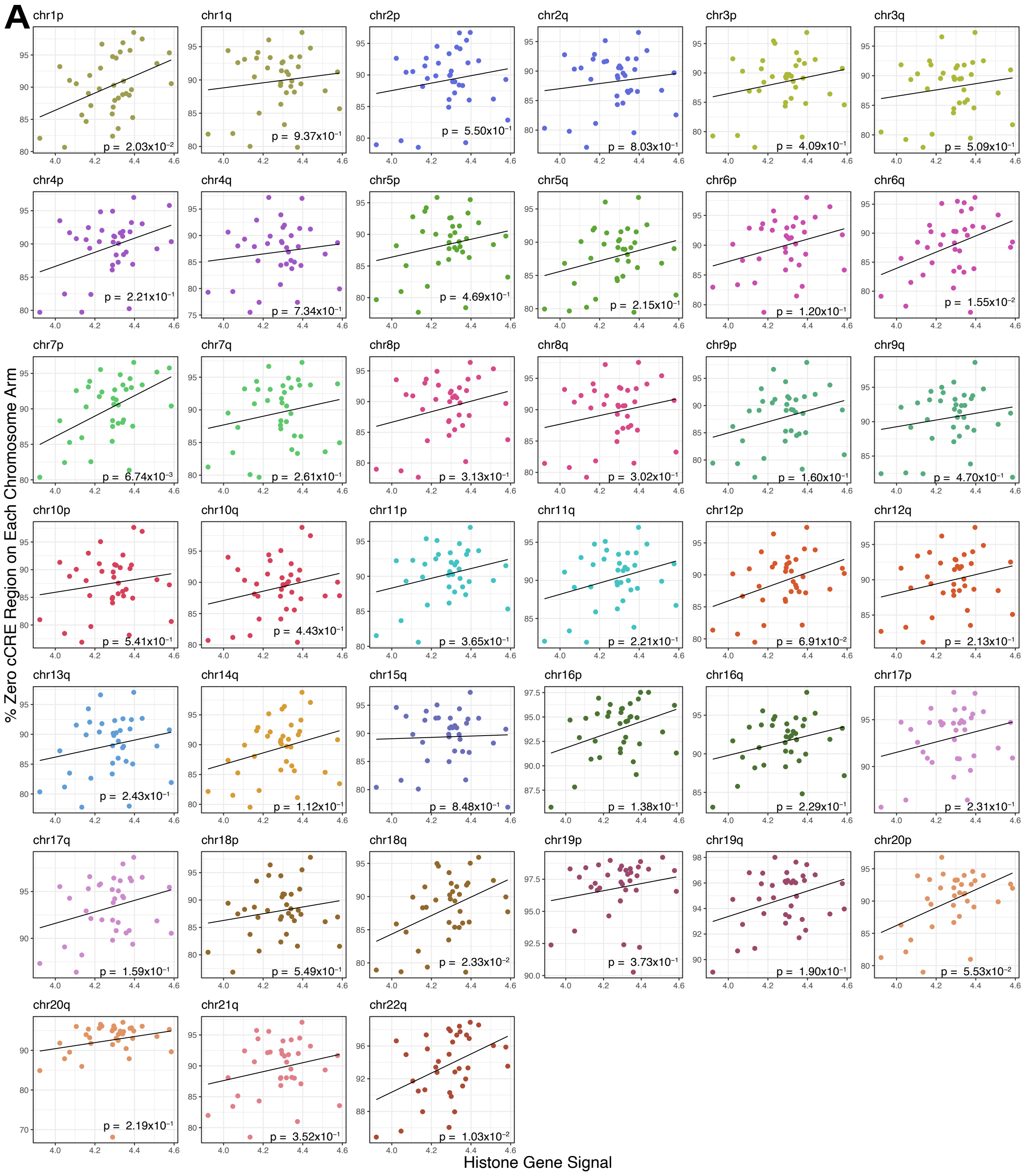
A
chr1p
p = 2.03x10^{-2}
chr1q
p = 9.37x10^{-1}
chr2p
p = 5.50x10^{-1}
chr2q
p = 8.03x10^{-1}
chr3p
p = 4.09x10^{-1}
chr3q
p = 5.09x10^{-1}
chr4p
p = 2.21x10^{-1}
chr4q
p = 7.34x10^{-1}
chr5p
p = 4.69x10^{-1}
chr5q
p = 2.15x10^{-1}
chr6p
p = 1.20x10^{-1}
chr6q
p = 1.55x10^{-2}
chr7p
p = 6.74x10^{-3}
chr7q
p = 2.61x10^{-1}
chr8p
p = 3.13x10^{-1}
chr8q
p = 3.02x10^{-1}
chr9p
p = 1.60x10^{-1}
chr9q
p = 4.70x10^{-1}
chr10p
p = 5.41x10^{-1}
chr10q
p = 4.43x10^{-1}
chr11p
p = 3.65x10^{-1}
chr11q
p = 2.21x10^{-1}
chr12p
p = 6.91x10^{-2}
chr12q
p = 2.13x10^{-1}
chr13q
p = 2.43x10^{-1}
chr14q
p = 1.12x10^{-1}
chr15q
p = 8.48x10^{-1}
chr16p
p = 1.38x10^{-1}
chr16q
p = 2.29x10^{-1}
chr17p
p = 2.31x10^{-1}
chr17q
p = 1.59x10^{-1}
chr18p
p = 5.49x10^{-1}
chr18q
p = 2.33x10^{-2}
chr19p
p = 3.73x10^{-1}
chr19q
p = 1.90x10^{-1}
chr20p
p = 5.53x10^{-2}
chr20q
p = 2.19x10^{-1}
chr21q
p = 3.52x10^{-1}
chr22q
p = 1.03x10^{-2}
% Zero cCRE Region on Each Chromosome Arm
Histone Gene Signal

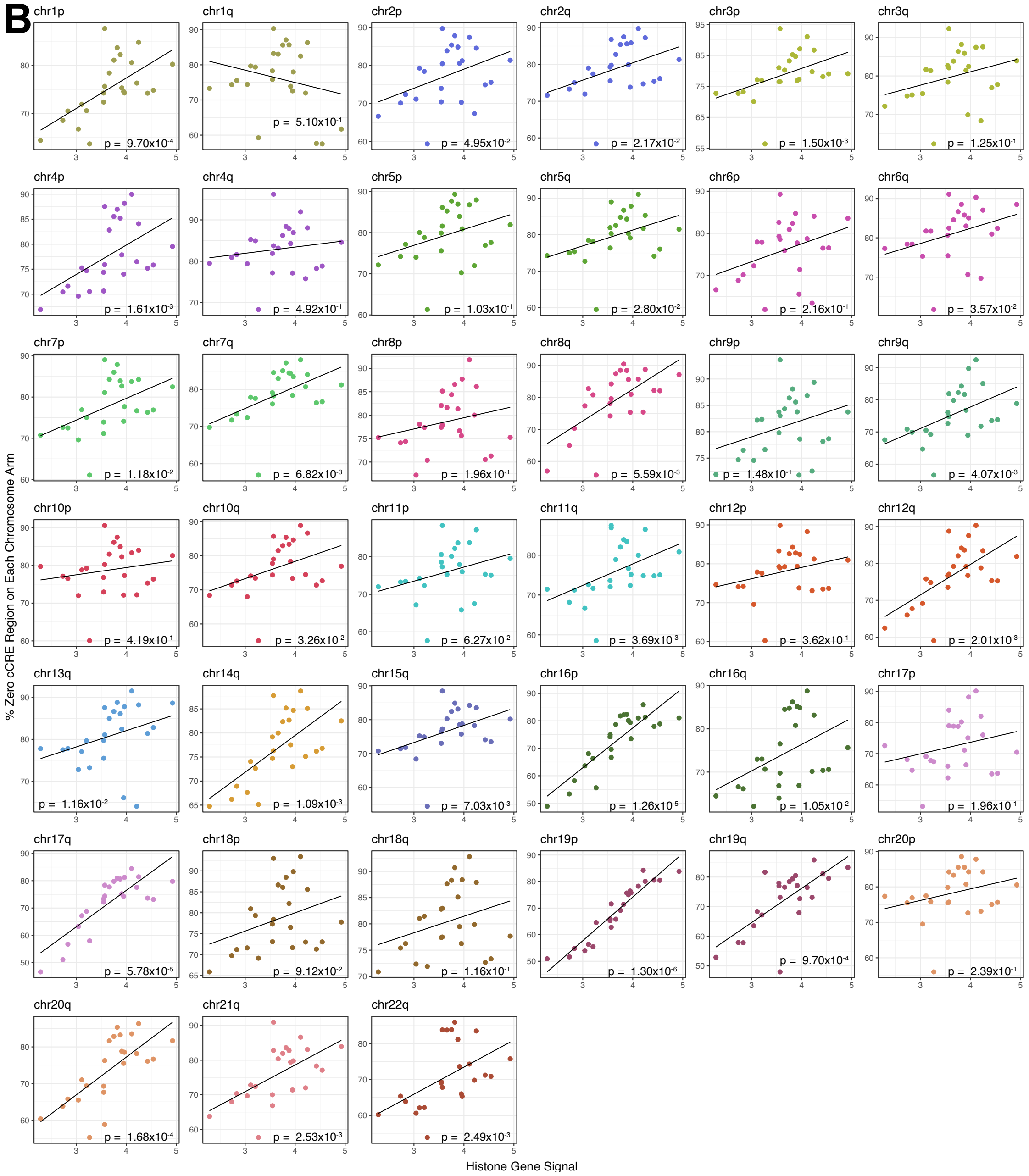

Whole-arm losses are more frequent than whole-arm gains in meningiomas as RNAPII signals increase on histone genes. To design a metric to quantify the relative frequencies of gains and losses for each of the 39 human autosomal arms (1p, 1q,...22q), we measured the percentage of cCRE regions with zero counts spanned by all cCREs on that arm for all meningiomas (**A**) and breast tumors (**B**). This yielded a single aneuploidy indicator value for each patient, whereby if the chromosome arm is lost, we should observe closer to 100% of cCRE regions with zero counts on that arm. We plotted this aneuploidy indicator on the *y*-axis against the aggregated RNAPII counts over the RC histone genes on the *x*-axis for that patient tumor, where the p-value for Spearman correlation is shown at the bottom of each panel. Figure 5 summarizes the correlation coefficients and significance levels on each arm. Excess of whole-arm losses (-1/2 dose) versus whole-arm gains (+3/2 dose) is counter-intuitive, as 1/500 newborns carry a trisomy but no newborn with a monosomy is known to have come to term.
